# Global subsoil organic carbon turnover times dominantly controlled by soil properties rather than climate

**DOI:** 10.1038/s41467-019-11597-9

**Published:** 2019-08-15

**Authors:** Zhongkui Luo, Guocheng Wang, Enli Wang

**Affiliations:** 1 CSIRO Agriculture & Food, GPO Box 1700, Canberra, 2601 ACT Australia; 2 LAPC, Institute of Atmospheric Physics, Chinese Academy of Sciences, Beijing, 100029 China; 3 Present Address: College of Environmental and Resource Sciences, Zhejiang University, Hangzhou, 310058 Zhejiang China

**Keywords:** Carbon cycle, Carbon cycle

## Abstract

Soil organic carbon (SOC) in the subsoil below 0.3 m accounts for the majority of total SOC and may be as sensitive to climate change as topsoil SOC. Here we map global SOC turnover times (*τ*) in the subsoil layer at 1 km resolution using observational databases. Global mean *τ* is estimated to be $1015_{729}^{1414}$ yr (mean with 95% confidence interval), and deserts and tundra show the shortest ($146_{114}^{188}$ yr) and longest ($3854_{2651}^{5622}$ yr) *τ* respectively. Across the globe, mean *τ* ranges from 9 (the 5% quantile) to 6332 years (the 95% quantile). Temperature is the most important factor negatively affecting *τ*, but the overall effect of climate (including temperature and precipitation) is secondary compared with the overall effect of assessed soil properties (e.g., soil texture and pH). The high-resolution mapping of *τ* and the quantification of its controls provide a benchmark for diagnosing subsoil SOC dynamics under climate change.

## Abstract (web summary)

The sensitivity of soil organic carbon (SOC) in subsoil (below 0.3 m) to climate change is poorly constrained. Here, the authors map global subsoil (0.3–1 m soil layer) SOC turnover times and find that temperature and in general climate effects are secondary to effects due to soil properties at both local and global scales—this now needs to be regarded for diagnosing subsoil SOC dynamics.

## Introduction

Soil organic carbon (SOC) represents the largest stock of organic carbon in the terrestrial biosphere^1,2^. Understanding how SOC dynamics respond to and provide feedbacks on climate change is vital for climate change mitigation as well as for sustaining ecosystem services important to agriculture intensification and biodiversity conservation^3–5^. The majority of studies paid more attention to the dynamics of SOC in the topsoil above 0.3 m^6^. Nonetheless, world subsoils below 0.3 m store two times more SOC than top 0.3 m soils^7^, which may also actively respond to climate change^6^ but is to date poorly understood. Main challenges are that subsoil SOC dynamics are more difficult to detect in situ. Although several site-specific studies have been conducted extending to deep soil layers^8,9^, it is difficult to extrapolate the results to other places due to the large spatial variability of soil conditions which may affect SOC decomposition differently.

The turnover time of SOC is a good indicator of soil carbon stability. By assessing the underlying drivers of SOC turnover time, we can gain insights into the dynamics of SOC under climate change and other soil disturbances, which otherwise are difficult to be obtained by conducting manipulation experiments. Most Earth-system models indeed rely on the regulation of climate-related variables (predominantly temperature and moisture) on turnover times of different carbon pools to predict soil carbon budgets both in the absence or presence of climate change^10–12^. Although several studies have estimated ecosystem carbon turnover times at the global scale^13^, subsoil SOC turnover times have not been explicitly quantified based on observational data, nor the mechanisms controlling the turnover. A detailed observation-based quantification of subsoil SOC turnover times and their association with climate and soil properties represents a key step towards reliable predictions of carbon cycle-climate feedbacks.

In a soil at the steady state, carbon turnover times (*τ*, yr) can be estimated as the ratio of total SOC pool size (SOC_total_) to carbon outputs or inputs (inputs = outputs under the steady state assumption). Inputs are equal to the net primary production (NPP, i.e., the difference between gross primary production and autotrophic respiration) that is allocated to the soil (NPP_soil_, i.e., belowground NPP) at the steady state, and thus *τ* can be estimated as: $\tau = \frac{{{\mathrm{{SOC}}}_{\mathrm{{total}}}}}{{{\mathrm{{NPP}}}_{\mathrm{{soil}}}}}.$ This calculation implicitly assumes SOC as a single homogenous cohort, and estimates the average residence time of carbon in the soil. In reality, however, few soils are at the strict steady state because of natural and anthropogenic disturbances (e.g., fire and land use change) and climate variability. The strict steady-state assumption has to be liberalized, because temporal monitoring of SOC with belowground NPP input presents a significant measurement challenge, making it difficult, if not impossible, to estimate real-time SOC turnover in situ. However, if the disturbances and/or environmental fluctuations are regular (e.g., management activities, and fire and drought regimes) and there is no long-term temporal directional trend, the soil could be considered to be in a quasi-steady state. Here we focus on *τ* in the 0.3–1 m soil layer in which SOC is much more stable than that in the topsoils. We call *τ* the apparent turnover time (Methods), which enables an evaluation of the spatial variability of SOC turnover times and an assessment of whether and how the turnover time correlates with climate and soil properties.

We estimate *τ* in the 0.3–1 m soil layer across the globe at the resolution of 0.0083° × 0.0083° (~1 km near the Equator) by combining global, spatially-explicit and observation-based SOC data in that layer [Harmonized World Soil Database (HWSD) version 1.2] and the Terra/MODIS NPP product^14^ at the same spatial resolution of SOC data. The NPP product reports the annual NPP from 2001 to 2015, and we calculate the average NPP over this period. Depending on the global biome distribution, a comprehensive global data set is compiled to fractionate total NPP to below- and above-ground NPP (Supplementary Data and Supplementary Table 1). The belowground NPP is further allocated to the subsoil (i.e., the 0.3–1 m soil layer) according to root biomass distribution in this layer in different biomes (Supplementary Table 2) by synthesising the ORNL DAAC dataset of global distribution of root profiles in terrestrial ecosystems^15,16^ (https://daac.ornl.gov/cgi-bin/dsviewer.pl?ds_id=660). The uncertainty in derived *τ* is estimated taking into account the uncertainties in NPP and its allocation to the 0.3–1 m soil layer.

## Results

### Spatial pattern of *τ* and its uncertainty

**Fig. 1:**
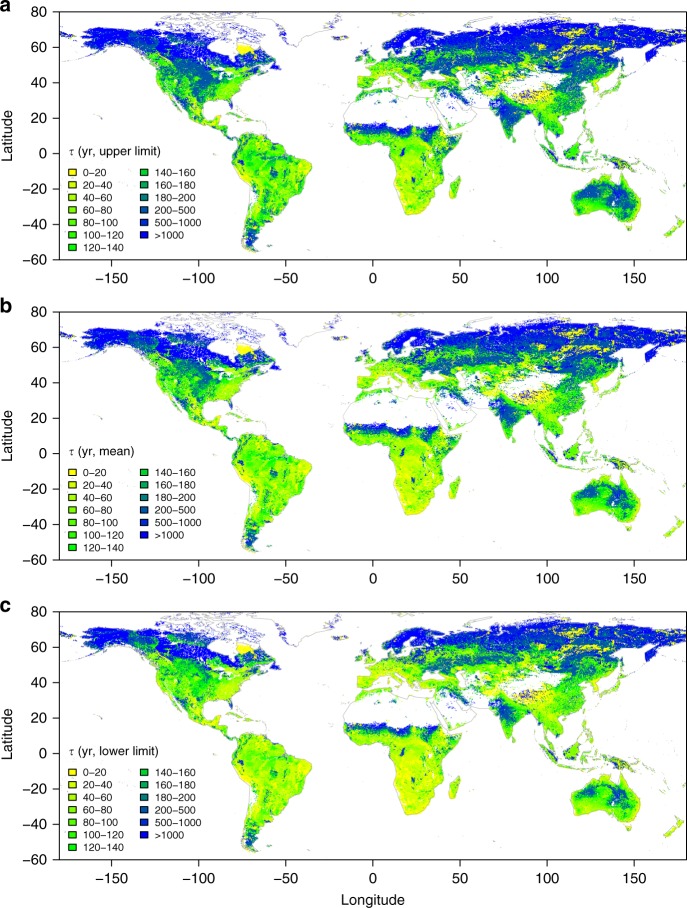
Spatial pattern of subsoil organic carbon turnover times. **a**, **b** and **c** respectively show the upper limit (i.e., the 97.5% quantile), mean, and lower limit (i.e., the 2.5% quantile) of subsoil (0.3–1 m) organic carbon turnover times based on 200 bootstrapping simulations considering uncertainty in carbon input in each pixel at the resolution of 0.0083°

**Fig. 2:**
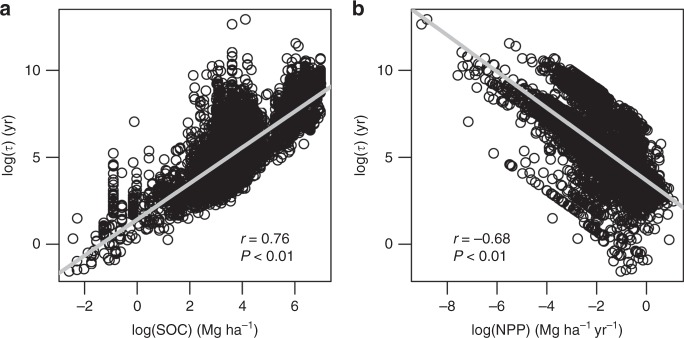
Correlation of turnover times with carbon stock and productivity. **a**, **b** The relationship of subsoil (0.3–1 m soil layer) organic carbon turnover times with soil organic carbon stock in that layer and net primary productivity allocated to that layer, respectively. This relationship is assessed based on data from 10,000 randomly sampled pixels across the globe. All data were natural log-transformed

Figure 1 shows the estimated *τ* in the 0.3–1 m soil layer and its uncertainty across the globe at the resolution of 0.0083°. Great spatial variability exists in *τ*, ranging from less than a decade to more than thousands of years (Fig. 1). For the upper limit of the 95% confidence interval (CI, see Methods section) of *τ*, its 5%, 25%, 50%, 75%, and 95% quantiles across the globe are 9, 78, 182, 609, and 6332 yr, respectively (Fig. 1a). These quantiles for mean *τ* are 7, 60, 137, 450, 4574 yr, respectively (Fig. 1b), and for the lower limit of *τ* are 5, 46, 104, 332, 3,303 yr respectively (Fig. 1c). It is general that *τ* increases with latitudes, particularly in the northern hemisphere (Fig. 1 and Supplementary Fig. 2). Another apparent pattern is that, *τ* positively correlates to SOC stock (Pearson’s correlation coefficient *r* *=* 0.76, Fig. 2a) and negatively correlates to NPP allocated to the 0.3–1 m soil layer (*r* = −0.68, Fig. 2b), as a result of the way *τ* is estimated (see details in Methods section).

Averaging across the globe, the global grand mean *τ* in the 0.3–1 m soil layer is $1015_{729}^{1414}$ yr (mean with its 95% CI, Table 1). Among biomes, *τ* generally reflects the temperature regime of biomes: biomes under colder climate have higher *τ* (Table 1). The grand mean *τ* is longest in two cold biomes: tundra ($3854_{2651}^{5622}$ yr) and boreal forests ($2017_{1466}^{2758}$ yr, Table 1). In other biomes, average *τ* is less than 1000 years, and two hot biomes—deserts and tropical/subtropical forests—have the shortest *τ* ($146_{114}^{188}$ and $168_{131}^{215}$ yr, respectively). In line with the latitudinal pattern of *τ* shown in Supplementary Fig. 2, these results suggest that temperature may play an important role in controlling *τ* at the global scale (see detailed assessment below).

**Table 1:** Mean turnover times (*τ*) of soil organic carbon in the subsoil (0.3–1 m soil layer) and its uncertainty in global biomes

Biome type	*τ* (yr)			Percentage uncertainty (%)
	Lower limit	Mean	Upper limit	
Global	729	1015	1414	59
Tropical/subtropical forests	131	168	215	51
Tropical/subtropical grasslands/savannas	603	799	1062	57
Temperate forests	156	195	246	46
Temperate grasslands	178	230	299	53
Mediterranean/montane shrublands	306	439	615	70
Boreal forests	1466	2017	2758	64
Tundra	2651	3854	5622	77
Deserts	114	146	188	51
Croplands	182	249	339	63

**Fig. 3:**
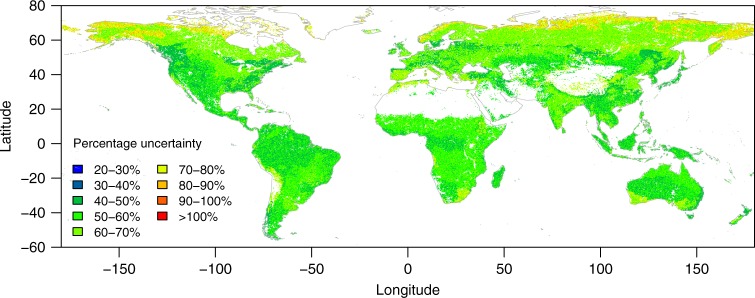
Percentage uncertainty of subsoil organic carbon turnover times. It is calculated as the percentage ratio of the difference between the upper and lower limits of the 95% confidence interval to the mean of 200 bootstrapping simulations considering uncertainty in carbon input in each pixel at the resolution of 0.0083°

Lower limit, mean and upper limit show the average of the results presented in Fig. 1a–c, respectively, while the percentage uncertainty shows the average of the results presented in Fig. 3.

### Percentage uncertainty

Figure 3 shows the percentage uncertainty (the ratio of the difference between the upper and lower limit of the 95% CI of *τ* in each pixel to the mean, see Methods section) in *τ* induced by NPP and its allocation to the subsoil. Similar to the spatial pattern of *τ* shown in Fig. 1, the percentage uncertainty of *τ* also shows great spatial variability, ranging from <30% to >100%. The 5%, 25%, 50%, 75%, and 95% quantiles of the percentage uncertainty across the globe are 45%, 51%, 58%, 65%, and 77%, respectively (Fig. 3). Averaging across the globe, the percentage uncertainty is 59% and comparable among biomes (Table 1). However, it is apparent that there is larger percentage uncertainty in higher northern latitudes, particularly in Tundra (>70% on average, Table 1 and Fig. 3).

### Controls of *τ* at the local scale

After mapping *τ* in subsoils across the globe, we assessed the relative influence (RI) of climate vs. soil properties on *τ* at local (~100 km × 100 km) and global scales. We focus on two climate variables [mean annual temperature (MAT) and precipitation (MAP)] and 10 soil variables [pH, clay, silt and sand content, electrical conductivity (ECE), sodicity (ESP), gypsum (CaSO_4_), calcium carbonate (CaCO_3_), total exchangeable bases(TEB), and base saturation (BS)] in the assessment.

**Fig. 4:**
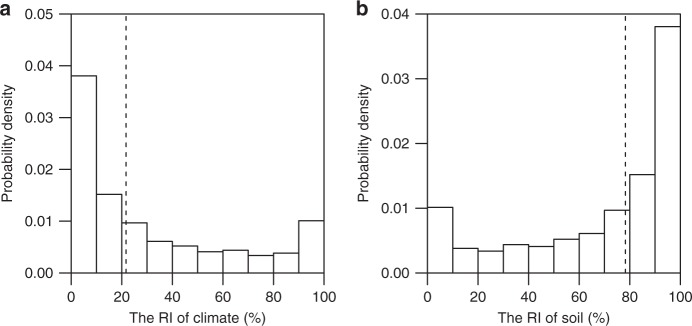
Relative importance of climate and soil at the local scale. **a**, **b** The relative importance of climate (including mean annual temperature and precipitation) and soil (including eight soil physiochemical properties) in controlling subsoil (0.3–1 m) organic carbon turnover times, respectively. The dashed line shows the weighted average relative importance of climate and soil across the globe (i.e., 22% for climate and 78% for soil), with weights as the model performance in each 100 × 100 km window

At the local scale, using localized boosted regression trees (BRT) taking into account potential interactions and non-linear relationships between variables (see Methods section), we find that the RI of climate (i.e., MAT and MAP together, see Methods section) is location-specific, ranging from zero to 100% (Fig. 4). Figure 4 shows the distribution of the RI of climate and soil. Averaging across the globe, climate represented by MAT and MAP together contributes 22% [i.e., model performance-weighted average RI (RI_w_), see Methods section] to the BRT model for *τ* (Fig. 4a), while soil represented by 10 soil variables together contributes 78% (Fig. 4b). In 23% of the area (at the scale of 100 km × 100 km grid) of the globe, MAT is the most important individual variable, followed by TEB (16%), clay (13%), BS (12%), sand (10%), MAP (8%), and other soil properties (Supplementary Fig. 3). We also quantified the fraction of locations (i.e., the 100 km × 100 km grids) where climate is more important than soil properties in controlling *τ* (i.e., RI_50_ in Supplementary Table 3), and find that climate is only more important in 26% of global locations (Supplementary Table 3).

**Fig. 5:**
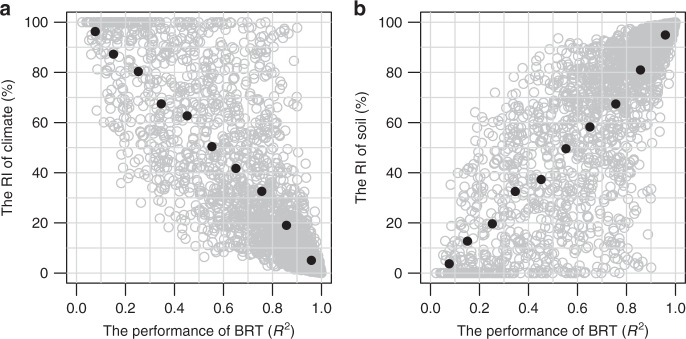
Relationship between model performance and the relative importance. **a**, **b** The relationship between the model performance (indicated by *R*^2^) and the overall relative importance (RI) of climate and soil variables, respectively. Grey open circles show the results in each 100 × 100 km window randomly selected for modelling. Black solid circles show the average for each group of *R*^2^ from 0 to 1 with 0.1 increment

It must be noted that the performance (*R*^2^) of the localized BRT varies substantially with the location of interest (Fig. 5 and Supplementary Fig. 4). The BRT model can on average explain 73% of the variances of *τ* at the local scale of 100 km × 100 km grid ranging from <5% to >95% across the globe (Fig. 5 and Supplementary Fig. 4). In order to detect the average pattern of the relationship between model performance and variable importance, we grouped *R*^2^ (i.e., model performance) into 10 groups from 0 to 1 with an increment of 0.1, i.e., [0–0.1), [0.1,0.2), ……, [0.9,1]. For each *R*^2^ group, we then calculated the average RI of both climate and soil. The result indicates that the average RI of climate decreases with model performance (Fig. 5a), while the average RI of soil increases with model performance (Fig. 5b). Overall, these results suggest that soil and climate had distinct overall effects on SOC turnover and other local scale variables may be also very important at the local scale.

### Controls of *τ* at the global scale

**Fig. 6:**
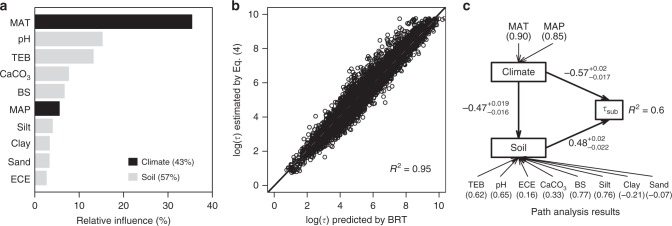
Controls over soil organic carbon turnover times at the global scale. **a** The relative influence (%) of predictors for the boosted regression tree (BRT) of turnover times. MAT mean annual temperature, MAP mean annual precipitation, pH soil pH, TEB the total exchangeable bases (i.e., the sum of exchangeable cations in the soil), ECE the electrical conductivity of the soil, BS base saturation, CaCO_3_ calcium carbonate. **b** The performance of the BRT in predicting estimated *τ*. The grey dashed line shows the 1:1 line. **c** Path analysis results on the direction and magnitude of the effects of latent variable climate (using MAT and MAP as indicators identified by the BRT) and soil (using pH, TEB, ECE, BS, CaCO_3_, clay, silt and sand content as indicators identified by the BRT) on turnover times. Numbers in the parentheses show the loading of the indicator to the latent variable, i.e., the correlation coefficents between the latent variables and the latent variables. See details in Methods section for the statistics

At the global scale, we randomly selected 10,000 pixels (at the resolution of 0.0083° × 0.0083°) to conduct the BRT (see Methods section). The BRT results suggest that MAT is much more important than MAP (36% vs. 5% in terms of relative influence, Fig. 6a). Soil pH and total exchangeable bases (15% and 13% in terms of their relative influence respectively) are the two most important soil variables. The relative individual influence of other soil properties is small (<10%), but the total relative contribution of soil variables to *τ* is 57%, overriding the 43% of climate (Fig. 6a). The BRT driven by the variables shown in Fig. 6a explains 95% of the variance in *τ* (Fig. 6b).

Path analysis at the global scale (see Methods section) further suggests that *τ* significantly and negatively correlates with climate (which is reflected by MAT and MAP), and significantly positively correlates with soil (which is reflected by eight significant soil variables identified by the BRT analysis shown in Fig. 6a) (Fig. 6c). In addition, we find that climate indirectly affects *τ* via its effect on soil properties (Fig. 6c). However, it is noteworthy that the overall performance of the path model (*R*^2^ = 0.6) in explaining the variability of *τ* is smaller than that of the BRT (*R*^2^ = 0.95, Fig. 6b, c), suggesting that more complex interactions and non-linear relationships than that considered by the path analysis may exist to regulate SOC turnover.

## Discussion

In this study we quantified spatially explicit turnover times of subsoil SOC at the global scale by integrating available observation-based databases. The global average turnover time of SOC in the 0.3–1 m soil layer was estimated to be $1015_{729}^{1414}$ yr. Previous studies mainly focus on topsoil layers^17^ or do not explicitly distinguish NPP allocation in different soil depths when estimating turnover times^11,18^. Using Earth system models (ESMs), for example, global average SOC turnover times in the top 1 m soil were estimated to be in the range of 10.8–39.3 yr^11^. This estimation considered SOC in the top 1 m soil as a cohort and did not take NPP allocation into account, making it difficult to infer SOC turnover times in subsoils. The credibility of those model estimations has been widely debated due to lack of observation-based verification^19,20^. Analysis of radiocarbon data sets from 157 soil profiles down to 1 m across the globe also challenged the estimates by ESMs and found that ESMs underestimate SOC turnover times by a factor of more than six^21^. Although less studies have assessed subsoil SOC turnover times, site-specific studies have indicated that subsoil SOC may be quite stable with turnover times of hundred and thousand years^22–24^, which is generally within the range estimated in this study. Our estimation of turnover times of subsoil SOC provides a reference to diagnose turnover times predicted by ESMs and by other empirical approaches.

To our knowledge, this study presents the first global maps of the spatial pattern of subsoil SOC turnover times and their uncertainty induced by carbon input into the subsoil. The results suggest that the uncertainty induced by uncertain carbon input is large (Table 1 and Fig. 3). This uncertainty is mainly due to the limited data availability and quality for estimating carbon input at the fine scale across the globe. In the approach presented in this study, a key parameter is the fraction of NPP allocated to subsoil. However, above- and belowground allocation of NPP remains one of the poorest understood attributes of terrestrial ecosystems. When further looking into the NPP allocation to different soil layers, the data becomes even scarcer and we have to indirectly infer NPP allocated to the 0.3–1 m soil using the information of root biomass distribution in the soil profile (see Methods section). Synthesizing the available datasets, we found that both plant NPP allocation to above- and belowground components (Supplementary Table 1) and root biomass distribution along the soil profile (Supplementary Table 2) are highly variable even in the same biome type, inevitably resulting in high uncertainty in the estimation of turnover times at the global scale. This variability reflects that plant community composition and structure and carbon allocation strategies adapt to local environmental conditions. In addition, accurate measurement of belowground processes such as belowground NPP allocation and rooting depth and biomass is also a big challenge, and the same process measured by different approaches also have large discrepancy^25,26^. These challenges make it difficult, if not impossible, to obtain large-scale measurements of rooting depth and belowground carbon allocation in situ. In addition, subsoil SOC turnover times are strongly associated with NPP allocated to the subsoil layer (Fig. 2b), implying that accurate partitioning of total NPP into the layer of interest is critical for robust estimation of SOC dynamics in different soil layers. To achieve this accuracy, we need innovative approaches to efficiently obtain data on belowground soil processes taking into account the high spatial variability of soil properties that regulate root growth and plant’s carbon allocation strategies.

At the global scale, our results demonstrate the larger overall effect of soil properties on subsoil SOC turnover than the effect of climate. This result may attribute to two reasons relating to carbon inputs to and outputs from the soil respectively. First, soil conditions have prominent effect on plant growth therefore the quantity and quality of soil carbon inputs. It is general that belowground resource availability (e.g., nutrients and water) has predominant effect on plant growth^27,28^, while the actual availability of those resources for plant growth is largely controlled by soil physiochemical environment. In terms of carbon outputs, soil physical (e.g., soil texture and porosity) and chemical properties (e.g., pH) directly determine carbon outputs via their effects on the transformation and stabilization of carbon inputs as well as the activity of decomposer community^29–31^. In addition, soil carbon can be protected from decomposition via occlusion with soil aggregates and binding with minerals^9,32,33^, and soil physiochemical characteristics determine the protective capacity of soil^34^. These physiochemical protection processes result in soil-dependent stabilization/destabilization of different soil carbon pools^34–36^. Overall, our BRT modelling and path analysis (Fig. 6) demonstrate the dominant role of soil physical and chemical characteristics in regulating SOC turnover at the global scale. We need novel approaches to derive metrics representing soil heterogeneity across the globe that can generally describe the effects of various soil properties on SOC dynamics.

At the local scale (i.e., 100 km × 100 km grid), there are some intriguing points about the importance of climate and soil, BRT model performance and their correlations (Figs. 4 and 5). On average, the model performance-weighted average importance (RI_w_) of climate at the local scale is much smaller than that at the global scale (22% vs. 43%), while the RI_w_ of soil at the local scale is higher than that at the global scale (78% vs. 57%). This result is reasonable as climate is generally much less variable at the local than at the global scale, while a greater part of soil variability observed at global scale can be observed at a finer scale. Considering that soil properties in HWSD are estimated by taxotransfer rules based on soil units (i.e., the same soil unit usually shares the same soil properties)^37,38^, the spatial variability of soil properties in HWSD at the local scale is underestimated at the local scale thereby the importance of soil properties. These results suggest that soil condition would be much more important than climate for regulating SOC turnover times at the local scale. The BRT model performance at the local scale, as well as the importance of climate and soil, is highly variable depending on the location. Areas with very low model performance imply that other local scale variables such as land use and topography^39,40^ may also have significant effect. In addition, we found that the performance of the BRT model has clear but distinct relationships with the RI of climate and soil, highlighting complex interconnections among soil, climate and other local scale variables in regulating SOC turnover times. Overall, our results suggest that the effect of soil properties and climate on local SOC turnover is highly variable depending on local conditions, although soil properties on average has greater effect than climate. Local information such as land use and management and terrain attributes are also required in order to more robustly predict SOC dynamics at the local scale.

Although the overall dominant role of soil properties, temperature exerts the most important individual influence on *τ* among all assessed variables, implying that subsoil SOC may be sensitive to temperature change and thus provides strong feedbacks on climate change under warming climate. However, our BRT modelling and path analysis imply that the temperature responses of SOC turnover may be highly variable, and strongly depend on the integrated effects of temperature on ecosystem processes that affect carbon inputs into and output from soil. Our local scale assessment, for example, indicates that temperature is not necessarily the most important variable everywhere (Supplementary Fig. 3). A recent site-specific study evidenced that subsoil SOC is very sensitive to warming and can significantly contribute to accelerated CO_2_ efflux under warming^6^. We must take care when extrapolating these site-specific findings to large scales or to other locations. As the effect of warming on some ecosystem processes may be site-specific, nonlinear over time or only manifest after a long time^41^, long-term observations will be critical for robust estimation of SOC balance under warming climate.

In this study, we have took particular care to the data sources and conducted a comprehensive literature review to synthesize available data sources, but there are still several limitations/uncertainties in the datasets propagating into the estimation of *τ* and the assessment on underlying controls. First, our data did not take into account any historical geological activities (e.g., volcanic explosion and glacier drift) that may dramatically change SOC dynamics. For these situations, SOC dynamics would be more relevant to those historical geological events rather than climate or soil conditions. However, this kind of geological activities is usually rare and may have limited effect on the spatial pattern of SOC turnover times across the globe. Second, we have to adopt the quasi-steady state assumption and do not have the global data to quantify the temporal variability of soil properties. In reality, some soil properties, particularly chemical variables like pH, may actively respond to external disturbance including human activities such as the utilization of synthetic fertilizers. If a system is not at the quasi-steady state, *τ* may be under- or over-estimated. Third, an important uncertainty source in this study is that a generic root distribution in the soil profile is used to estimate carbon allocation to the subsoil for the same biome type. Although this simplification is required for the application at the global scale and its consequences on turnover time estimation has been considered via uncertainty assessment, it has to be noted that it does not explicitly consider the potential effects of local soil environment such as soil depth and other constraints that affect root distribution in the soil profile and thus the estimation of *τ*. Fourth, we do not consider the effect of potential uncertainties in subsoil SOC stocks on the estimation of SOC turnover times. This uncertainty of SOC in the HWSD database may be large as the strong correlation between SOC stock and its turnover time (Fig. 2a), and is particularly important in high northern latitudes (e.g., tundra and boreal forest areas) as the HWSD relies very few observations for SOC estimation in these areas^42–44^. Although the HWSD database reports soil information at the resolution of 1 km, at last, it is generated based on soil units^37,38^. That means adjacent 1 km × 1 km pixels may have the same soil unit and thus share the same soil properties including SOC, which would largely underestimate the spatial variability of soil properties. However, NPP and climate data are not based on soil units, and are produced at the resolution of 1 km. This kind of resolution mismatch between different databases may bias the estimation of SOC turnover times as well as the relative importance of climate and soil properties. We acknowledge that all these limitations should be overcome to provide more robust predictions on *τ* and its controls, which is particularly important for regions experiencing significant human-dominant land management and shift of land use and cover.

In conclusion, we have presented the first spatially explicit quantification of SOC turnover times in world subsoils (0.3–1 m) using observation-based databases at the resolution of 0.0083° and by integrating published datasets. We find that SOC turnover time in the 0.3–1 m soil layer on average is ~1000 yr ranging from less than a decade to more than thousand years across the globe, providing a reference to judge the credibility of predictions of current Earth-system models^11,17^ as well as to compare with estimations using other empirical approaches. Our results demonstrate that the overall effect of climate (including temperature and precipitation) on subsoil SOC turnover is secondary compared with the overall effect of soil properties at both global and local scales, albeit the great variability of the importance of climate at the local scale. The effects of individual soil variables are relatively small, but they work together involving complex interactions and non-linear relationships with each other as well as with climate to regulate SOC dynamics^9,32,45,46^. For this reason, soil physiochemical conditions may significantly shape the direction and magnitude of the response of subsoil SOC dynamics to climate change, and thus should be explicitly considered in order to reliably predict soil carbon-climate feedbacks. In conflict with our findings on the dominant importance of soil properties in controlling subsoil SOC turnover, prevailing soil carbon and Earth system models are primarily driven by climate and built upon knowledge of topsoil SOC dynamics^11^. Here we argue that a specific subsoil module should be developed to properly simulate subsoil SOC dynamics taking into account the potential distinct sub- and top-soil environment, otherwise the subsoil SOC dynamics would be unreliably predicted. However, in all assessed variables, we find that temperature in general is the most influential individual variable on subsoil SOC turnover, highlighting the potential sensitivity of subsoil SOC to warming. The mapping of SOC turnover times and their uncertainty and the assessment on their drivers will facilitate screening regions sensitive to climate change and guide site-specific policy-making for effective carbon management.

## Methods

### SOC data and soil properties

The Harmonized World Soil Database (HWSD) version 1.2 (http://www.fao.org/soils-portal/soil-survey/soil-maps-and-databases/harmonized-world-soil-database-v12/en/) reports global SOC content in the top 0–0.3 m (topsoil) and 0.3–1 m (subsoil) soil at the resolution of 30 arc-second (i.e., ~0.0083° degrees or ~1 km near the Equator). In brief, the HWSD is generated based on measurements of thousands of soil profiles across the globe^37,38^. For each ~1 km grid, a soil unit was determined first. Then, if measured data of a typical soil property is available for the soil unit, it was derived directly based on the measured data attached to the soil unit; if not, taxonomy-based pedotransfer rules were used for estimation^37,38^. This database combines existing regional and national updates of soil information worldwide and represents the most comprehensive soil database to present. With the SOC content, a series of other soil physical and chemical properties are also reported for both topsoil and subsoil in each pixel (0.0083° × 0.0083°).

In this study, we focus on subsoil SOC. In each pixel, we estimated total SOC stocks (SOC, kg C m^–2^) in the subsoil as: $SOC = \frac{{OC}}{{100}} \cdot D \cdot BD \cdot \left( {1 - \frac{G}{{100}}} \right),$ where *OC* is the SOC content reported as the percentage of soil weight in the HWSD, *D* the thickness of the soil layer being considered (i.e., 0.7 m), *BD* the soil bulk density, and *G* the gravel content reported as the percentage of soil volume. Except those soil variables for calculating SOC stocks, we also extracted other soil properties in the subsoil including pH, clay, silt and sand content, electrical conductivity (ECE), sodicity (ESP), gypsum (CaSO_4_), calcium carbonate (CaCO_3_), total exchangeable bases (TEB) and base saturation (BS). The association of SOC turnover times with these variables were assessed (see details in the subsection: Drivers of *τ* at the local and global scales).

### Net primary productivity (NPP) and biomes

The NPP data produced by the Numerical Terradynamic Simulation Group (NTSG)/University of Montana (UMT) was obtained^14^. This NPP product was generated by analysing satellite data from the moderate-resolution imaging spectroradiometer (MODIS). The global MODIS NPP algorithm^14,47^ was used to estimate annual NPP from 2001 to 2015. Basic inputs into the algorithm include the fraction of photosynthetically active radiation and leaf area index data from the MODIS sensor collected at 8-day time interval and at the resolution of 1 km. The average annual NPP in the period 2001–2015 was calculated.

The amount of NPP allocated to the subsoil (i.e., NPP allocated to the 0.3–1 m soil layer) is a key variable for the estimation of SOC turnover times. In this study, we assumed that the same biome shares the same allocation strategy of total NPP. First, we generated a global map of biome types by merging two land cover maps: the MODIS land cover map^48^ and the WWF (World Wildlife Fund) map of the Terrestrial Ecoregions of the World^49^ (https://www.worldwildlife.org/publications/terrestrial-ecoregions-of-the-world). The two maps are aggregated to generate a biome map having the same resolution of SOC and NPP databases and including nine biomes: tropical/subtropical forests, tropical/subtropical grasslands/savannas, temperate forests, temperate grasslands, Mediterranean/montane shrublands, boreal forests, tundra, deserts, and croplands.

### The allocation of NPP

In order to calculate *τ*, NPP has to be allocated into the subsoil. To do so, we first compiled a comprehensive database of NPP and its above- and belowground allocation by conducting a thorough literature search. A total of 471 observations of *f*_BNPP_—the fraction of belowground NPP in total NPP—were obtained from 54 papers in which *f*_BNPP_ was directly reported or can be calculated (Supplementary Data 1). In addition, the ORNL DAAC NPP data collection (https://daac.ornl.gov/cgi-bin/dataset_lister.pl?p=13) was also screened, and the data sets that enable the calculation of *f*_BNPP_ were included. Finally, we obtained a NPP database with 848 field observations of *f*_BNPP_ covering all the nine biomes across the globe (Supplementary Fig. 1 and Data 1).

Based on the estimated *f*_BNPP_ (Supplementary Data 1 and Table 1), we can partition total NPP (i.e., the average NPP during the period 200–2015) to aboveground and belowground NPP based on biome types (Supplementary Table 1)^27^. The belowground NPP was then partitioned into the 0.3–1 m subsoil based on the vertical root biomass distribution in the soil profile (Supplementary Data 2)^16^. An empirical model (i.e., the logistic dose–response curve) was used to interpolate root distribution in the soil profile:^15^

1 $$r_D = \frac{{R_{max}}}{{1 + \left( {\frac{D}{{D_{50}}}} \right)^c}},$$

where *r*_*D*_ is the total amount of roots above soil depth *D* (m), *R*_max_ is an estimate for the total amount of roots in the whole soil profile, *D*_50_ is the depth (m) at which *r*_*D*_ = 0.5·*R*_max_, and *c* is a dimensionless shape parameter and is calculated as: $c = \frac{{ - 1.27875}}{{log_{10}D_{95} - log_{10}D_{50}}}$, where *D*_95_ is the depth (m) at which *r*_*D*_ = 0.95·*R*_*max*_. According to Eq. (1), the fraction of roots in the 0.3–1 m soil layer (fr_0.3–1_) can be estimated as:

2 $$fr_{0.3 - 1} = \frac{{r_1}}{{R_{max}}} - \frac{{r_{0.3}}}{{R_{max}}} = \frac{1}{{1 + \left( {\frac{1}{{D_{50}}}} \right)^c}} - \frac{1}{{1 + \left( {\frac{{0.3}}{{D_{50}}}} \right)^c}}.$$

Schenk and Jackson^15^ had complied a comprehensive dataset of root distribution in 564 soil profiles across the globe (this data is publically available at (https://daac.ornl.gov/cgi-bin/dsviewer.pl?ds_id=660), Supplementary Fig. 1) to train the interpolation model, and also compared different interpolation models and found that the logistic dose–response curve is the most robust. Here we used this root profile dataset including the interpolated *D*_50_ and *D*_95_ by Schenk and Jackson to estimate fr_0.3–1_ in each soil profile. The Schenk and Jackson dataset does not include crops. For crops, we used the estimation of *D*_50_ reported by Fan et al.^50^ who used the same logistic dose–response curve to interpolate the distribution of root profiles of different crops by synthesising 96 root profiles of crops across world croplands. However, the data of the 96 crop root profiles used by Fan et al. is not available for us, and thus we cannot directly estimate the variance of crop root distribution as that in other biomes (Supplementary Table 2). When conducting uncertainty assessment (see the subsection: Estimation of SOC turnover times and its uncertainty), we reassigned the standard error of *D*_*50*_ as 10% of its mean. The estimated *fr*_*0.3-1*_ based on those global databases was then grouped into the nine biomes. Coupling with the estimated *f*_*BNPP*_, the amount of total NPP allocated to the 0.3–1 m soil layer (BNPP_0.3-1_) can be calculated as:

3 $${\mathrm{{BNPP}}}_{0.3 - 1} = {\mathrm{{NPP}}} \cdot f_{\mathrm{{BNPP}}} \cdot fr_{0.3 - 1}.$$

This estimation of BNPP_0.3–1_ takes into account the root residues and exudates/rhizodeposition, but assumes that net vertical physical transportation of carbon (e.g., dissolved organic carbon washed into the subsoil from upper layers and/or leached into deeper layers) is neutral. We acknowledge that information on the movement of dissolved organic carbon in the soil profile may improve the relevant estimations.

### Estimation of SOC turnover times (*τ*) and its uncertainty

SOC turnover time (*τ*) is the average time between when a carbon atom enters the soil until it exists the soil. For a soil at the steady state, carbon output equals to input and thus *τ* can be estimated as the ratio of the total SOC pool size to carbon output or input (*C*_in_): $\tau = \frac{{\mathrm{{SOC}}}}{{C_{\mathrm{{in}}}}}.$ Under the steady state assumption, the *C*_in_ can be estimated as the amount of NPP allocated to the soil if assuming neutral vertical physical transportation of organic carbon. In this study, we focus on *τ* in the 0.3–1 m subsoil layer as the steady state assumption would be more valid in this layer, and *τ* can be estimated as:

4 $$\tau = \frac{{{\mathrm{{SOC}}}_{0.3 - 1}}}{{{\mathrm{{BNPP}}}_{0.3 - 1}}} = \frac{{{\mathrm{{SOC}}}_{0.3 - 1}}}{{{\mathrm{{NPP}}} \cdot f_{\mathrm{{BNPP}}} \cdot {\mathrm{{fr}}}_{0.3 - 1}}}.$$

Although we can estimate *τ* in the topsoil, topsoils may more frequently suffer from disturbances and aboveground plant residues may be also a large contributor to the overall carbon input into the topsoil, resulting in the difficulty to adopting the steady state assumption. We note some characteristics of *τ* and the four variables in Eq. (4). First, previous studies tracing the cycling of carbon isotopes estimated that *τ* may range from several decades to centuries depending on ecosystems even in the topsoil^21,51^. That means, in Eq. (4), the magnitude of SOC is much greater than the magnitude of BNPP_0.3–1_, and thus *τ* will be more sensitive to the denominator BNPP_0.3–1_. Second, SOC would be much more stable at the inter-annual scale compared with NPP. For these reasons, we focused on the uncertainty in *τ* induced by BNPP_0.3–1_.

It is straightforward that there are three uncertainty sources in BNPP_0.3–1_ induced by NPP, *f*_BNPP_, and fr_0.3–1_, respectively. For the uncertainty in NPP, there is no concrete assessment to date on the uncertainty in the MODIS NPP product because of the limited available field data for validation and the mismatch in scales between ground-based NPP measurements and the resolution (1 km) of the NPP product^14,47^. Here, we used an empirical Monte Carlo approach to bring the uncertainty in NPP into the estimation of BNPP_0.3–1_. First, we assumed a normal distribution of NPP in each 1 km pixel with a mean estimated by the 15-year MODIS NPP product in that pixel and standard deviation (SD) assigned as the 10% of the mean, i.e., NPP~N(mean, SD). Then, 200 Monte Carlo samples were randomly drawn from the normal distribution to obtain 200 NPP estimates. At the same time, a non-parametric bootstrapping approach was used to quantify the uncertainty in *τ* induced by the other two variables via randomly sampling 200 bootstrap combinations of these variables. Specifically, 200 bootstrap estimates of *f*_BNPP_ and fr_0.3–1_ were also generated by randomly sampling the derived data described above, respectively, depending on the biome type which the pixel belongs to. For fr_0.3–1_ in croplands, it was estimated by randomly sampling a normal distribution of *D*_50_ with the mean reported by Fan et al. and the SD as 10% of the mean. Putting these sampling estimates together, we obtained 200 ensembles of NPP, *f*_BNPP_, and fr_0.3–1_ and thus 200 estimates of *τ* for each pixel. Based on the 200 estimates of *τ*, then we calculated the mean and 95% confidence intervals (CI, i.e., 2.5% and 97.5% quantiles of the 200 bootstrapping samples) of *τ*. The 95% CI represents the uncertainty in *τ* induced by carbon input (i.e., NPP·*f*_BNPP_·fr_0.3–1_).

Although subsoils would be more stable than topsoils, we acknowledge that rarely do soils stay at the strict steady state and *τ* estimated by Eq. (4) may suffer from bias in reality. We have to relax the strict steady state assumption. Here, we call *τ* the apparent turnover time and interpret it as an emergent diagnostic according to ref. ^13^. The estimation of *τ* in the subsoil makes it possible to quantify the spatial variability of SOC turnover times in the subsoil at the global scale, providing a reference and benchmark for assessing whether, how, where and to what extent the SOC turnover in the subsoil may respond to climate and management changes. Based on the best available information, our approach provides the finest observation-derived estimation of global SOC turnover times in the subsoil at the resolution of 0.0083°. The estimations can be aggregated to and used for uncertainty assessment at coarser scales. The estimated *τ* can be obtained by contacting the authors and all other data sets are publicly available.

### Climate data

In order to test the correlation between SOC turnover times and climate variables, we obtained the global mean precipitation and temperature reported in WorldClim version 2.0 (http://worldclim.org/version2)^52^. The WorldClim version 2 reports the mean annual temperature (MAT) and precipitation (MAP) for the period 1970–2000 at the same spatial resolution of the HWSD and NPP data. The time frame of the climate data is not consistent with that of the NPP data. However, this is the most comprehensive data sets we can obtain, and its resolution is also consistent with the HSWD and NPP databases, facilitating the assessment of the effect of climate on SOC turnover times. As we focus on the long-term average conditions of climate, the effect of this discrepancy between the time frames would have limited effect on the relevant statistical results.

### Drivers of *τ* at local and global scales

In this study, SOC turnover times (*τ*) are calculated based on SOC stock and NPP. As such, any factors potentially correlated to or influencing SOC and/or NPP may to some extent affect SOC turnover times. Here, we assessed the relative importance (RI) of climate (i.e., MAT and MAP) and soil properties (i.e., the 12 soil variables collated from the HWSD) in controlling *τ* (focusing on mean *τ*) at local and global scales. For each 0.0083° × 0.0083° pixel for the local scale assessment, we trained a BRT model using a common 10-fold cross-validation strategy^8,53^ based on the data in a window of 101 by 101 pixels (i.e., the window size is ~100 × 100 km) centred at the target pixel to explore the local variability of the relationships between covariates (i.e., climate and soil properties) and the response variable τ. Considering that not all pixels have data, the BRT model was only trained if the number of pixels with data in a window is >50. The BRT analysis involves a type of data-mining (machine-learning) algorithm that combines the advantages of a regression tree (decision tree) algorithm and boosting^53^. It can analyse different types of variables and interaction effects between variables and are applicable to nonlinear relationships, and identify the RI of predictor variables (i.e., climate and soil variables in this study). The RI of individual variables for climate and soil properties are summed respectively to indicate the overall relative importance of climate and soil. Using this approach, we intended to yield a global map of the RI of climate and soil properties in controlling local *τ* variability at the same resolution of *τ*. However, the computing time required to complete this job is huge (4 months assuming 1000 modern computer cores), making it impossible to generate such map. For this reason, we used a Monte Carlo approach by randomly selecting 10,000 ~100 × 100 km windows to conduct the BRT. Based on the BRT results at the local scale, we calculated the weighted average relative importance (RI_w_) of both climate and soil at the local scale across the globe as well as in the nine biomes:

5 $$RI_w = \frac{{\mathop {\sum }\nolimits_{i = 1}^n \left( {RI_i \cdot R_i^2} \right)}}{{\mathop {\sum }\nolimits_{i = 1}^n \left( {R_i^2} \right)}},$$

where *RI*_*i*_ and $R_i^2$ are the relative importance of climate (or soil) and model performance (i.e., the coefficient of determination *R*^2^ in this study) in the *i*^th^ Monte Carlo simulation, respectively. For the global scale assessment, we randomly selected 10,000 pixels with the relevant data to train a BRT model.

In addition to the relative and overall importance of climate and soil identified by the BRT analysis, we used path analysis to quantitatively partition the direct from indirect effects of climate and soil on *τ*. We conducted the path analysis using the data from the 10,000 randomly selected pixels focusing on the global scale results. To simplify the path model, the significant variables identified by the BRT analysis were used to indicate two latent variables: climate and soil. For the latent variable climate, the two potential indicators were: MAT and MAP. Soil geochemical properties including pH, soil texture (i.e., clay, silt and sand content), ECE, BS, CaCO_3_, CaSO_4_, ESP, and TEB reported in the HWSD database were considered as potential indicators for the latent variable soil. We considered the following potential paths in a hypothesis-oriented path model. First, we hypothesized that both latent variables have direct effect on *τ*. Second, climate may also indirectly affect *τ* through its effect on soil properties. The partial least squares (PLS) approach was used for the path analysis^54^. In the PLS path analysis, the loading of each indicator variable is the key to estimate latent variable scores and calculated as the correlation between a latent variable and its indicators. An iterative algorithm is used to estimate the loadings until the convergence of the loadings is reached to maximize the explained variance of the dependent variables (both latent and indicator variables). A non-parametric bootstrapping (200 resamples in this study) was used to estimate the precision of the PLS parameter estimates. The 95% bootstrap confidence interval was used to judge that whether the estimated path coefficients are significant. To ease interpretation, if an indicator has a negative loading, its opposite was used in the model to ensure a positive loading, and all indicators were standardized. The BRT analysis and path analysis were performed using the package gbm and dismo, and plspm, respectively, in R 3.3.1 (R Core Team 2016).

## Supplementary information

Supplementary Information

Peer Review File

Description of Additional Supplementary Files

Supplementary Data 1

Supplementary Data 2

Supplementary Software 1

## Data Availability

All the data used in this study can be publically accessed through the correspondingly provided website links in the text. The NPP allocation data is provided as a supplementary data. An R script is provided as Supplementary Software 1 to showcase the procedure of fitting BRT models. The estimated turnover times (Fig. 1) and their uncertainty (Fig. 3) can be accessed at (10.6084/m9.figshare.8194652). All other relevant data are available from the corresponding authors.
