# Supplementary material for: Global subsoil organic carbon turnover times dominantly controlled by soil properties rather than climate
Source: Nat Commun. 2019 Aug 15;10:3688. doi: 10.1038/s41467-019-11597-9 (PMC6695437; doi:10.1038/s41467-019-11597-9)
Supplement: Supplementary file 1 — Supplementary Information [file 41467_2019_11597_MOESM1_ESM.pdf]

Global subsoil organic carbon turnover times dominantly controlled by soil properties rather than climate

Luo et al.

Supplementary Figures

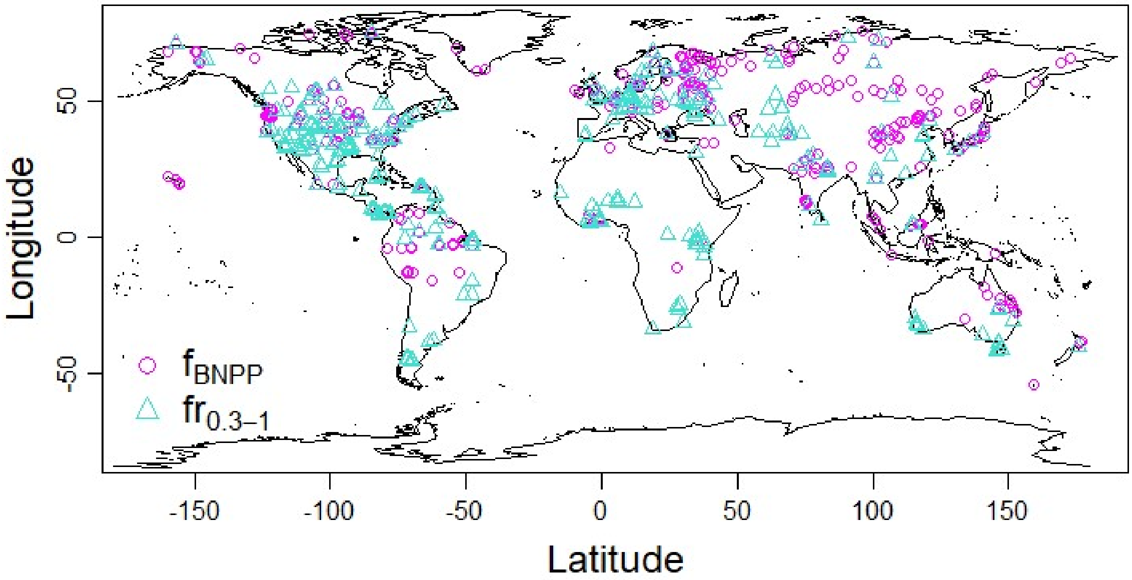

Supplementary Figure 1. Location of field measurements of *f_{BNPP}* and *$fr_{0.3\text{-}1}$*. f_{BNPP} and $fr_{0.3\text{-}1}$ are the fraction of belowground NPP in total NPP and the fraction of root biomass distribution in the 0.3-1 m soil layer, respectively.

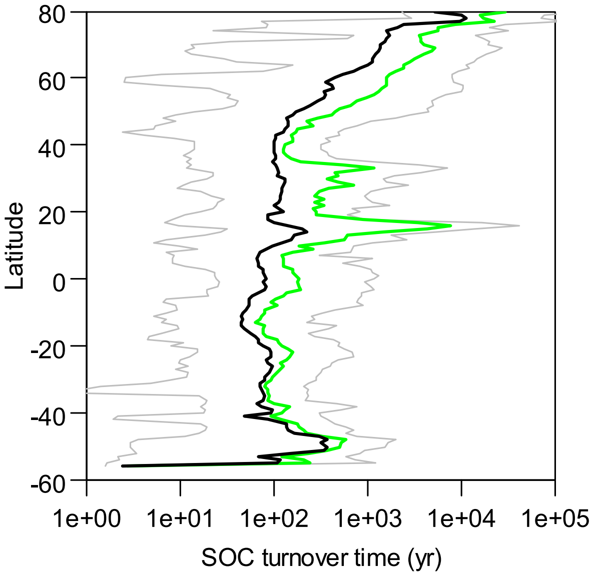

Supplementary Figure 2. Latitudinal patterns of subsoil (0.3–1 m) SOC turnover times. Black and green lines shows the 50% quantile (i.e., median) and average respectively, while left and right grey lines show the corresponding 2.5% and 97.5% quantiles, respectively.

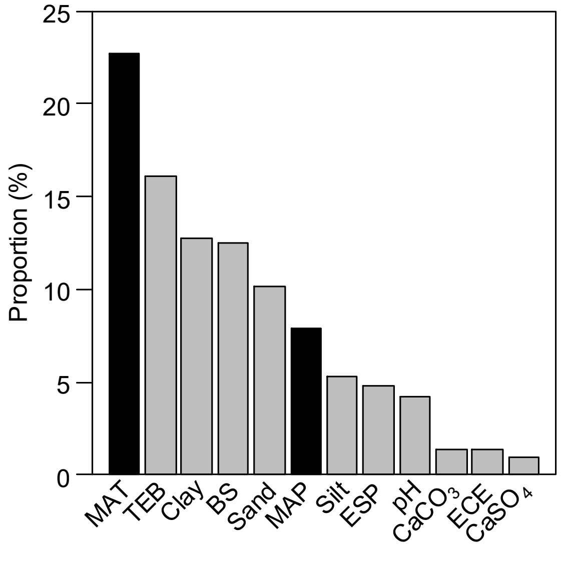

Supplementary Figure 3. The proportion of the variable that is the most important for subsoil SOC turnover at the local scale (~100×100 km) across the globe. A value of 10% for a typical variable means that the variable is the most important one in 10% of pixels across the globe. See Methods for the abbreviation of the variables. Black and grey bars show climate and soil variables respectively.

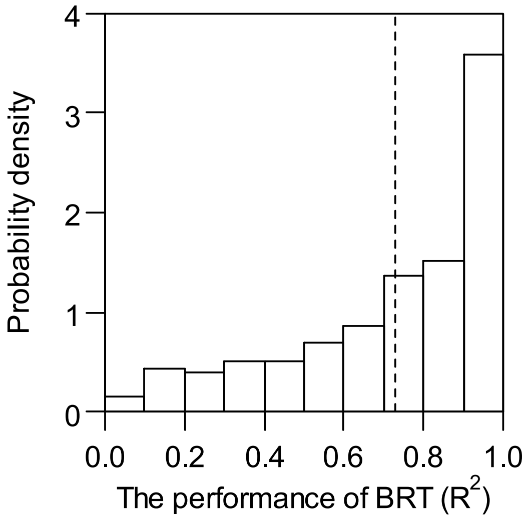

Supplementary Figure 4. The distribution of the performance of BRT model at local scale across the globe. The dashed line shows the global average of model performance.

Supplementary Tables

Supplementary Table 1. Below-ground allocation of net primary production (NPP, f_{BNPP}) in nine biomes. See detailed data in the Supplementary Data 1.

Biome type	f_{BNPP}			n
	Q2.5	Mean/Median	Q97.5	
Global	0.074	0.41/0.39	0.92	848
Tropical/subtropical forests	0.074	0.32/0.32	0.61	164
Tropical/subtropical grasslands/savannas	0.12	0.37/0.36	0.62	25
Temperate forests	0.084	0.34/0.29	0.79	186
Temperate grasslands	0.2	0.63/0.60	0.93	121
Mediterranean/montane shrublands	0.21	0.68/0.70	0.99	27
Boreal forests	0.045	0.32/0.27	0.75	178
Tundra	0.074	0.43/0.43	0.87	72
Deserts	0.27	0.67/0.63	0.96	53
Croplands	0.086	0.27/0.23	0.59	22

Q2.5, Mean, Median, and Q97.5 are the 2.5%, average, 50%, and 97.5% quantiles, respectively. *n*, the sample size.

Supplementary Table 2. The fraction of root biomass distribution in the 0.3-1 m soil layer ($fr_{0.3-1}$) in different biomes. See detailed data in the Supplementary Data 2.

Biome type	$fr_{0.3-1}$			n
	Q2.5	Mean/Median	Q97.5	
Global[a]	0.014	0.22/0.18	0.61	564
Tropical/subtropical forests	0.0087	0.18/0.16	0.53	97
Tropical/subtropical grasslands/savannas	0.047	0.29/0.25	0.64	65
Temperate forests	0.056	0.28/0.26	0.61	133
Temperate grasslands	0.016	0.16/0.11	0.56	109
Mediterranean/montane shrublands	0.037	0.19/0.14	0.51	29
Boreal forests	0.0029	0.11/0.097	0.30	43
Tundra	0.0060	0.053/0.048	0.12	24
Deserts	0.033	0.34/0.30	0.69	64
Croplands[b]	-	0.25/-	-	96

Q2.5, Mean, Median, and Q97.5 are the 2.5%, average, 50%, and 97.5% quantiles, respectively. *n*, the sample size. [a] Croplands are excluded. [b] Adopted from Fan et al. (2016).

Supplementary Table 3. The relative influence of climate in controlling subsoil SOC turnover times (τ) in global biomes.

Biome type	RI for τ (%)	
	RI_{50}	RI_w
Global	26	22
Tropical/subtropical forests	18	15
Tropical/subtropical grasslands/savannas	27	20
Temperate forests	24	19
Temperate grasslands	26	22
Mediterranean/montane shrublands	40	30
Boreal forests	20	12
Tundra	39	26
Deserts	34	27
Croplands	22	17

RI_{50}, the fraction of locations (i.e., 100 km × 100 km window) where climate is more important than soil properties; RI_w, the model performance-weighted average RI of climate for all soils.

References for Supplementary Data 1

An, J. Y., Park, B. B., Chun, J. H., & Osawa, A. (2017). Litterfall production and fine root dynamics in cool-temperate forests. PloS one, 12(6), e0180126.

Aragão, L. E. O. C., Malhi, Y., Metcalfe, D. B., Silva-Espejo, J. E., Jiménez, E., Navarrete, D., ... & Anderson, L. O. (2009). Above-and below-ground net primary productivity across ten Amazonian forests on contrasting soils. Biogeosciences, 6(12), 2759-2778.

Bolinder, M. A., Janzen, H. H., Gregorich, E. G., Angers, D. A., & VandenBygaart, A. J. (2007). An approach for estimating net primary productivity and annual carbon inputs to soil for common agricultural crops in Canada. Agriculture, Ecosystems & Environment, 118(1-4), 29-42.

Bond-Lamberty, B., Wang, C., & Gower, S. T. (2004). Net primary production and net ecosystem production of a boreal black spruce wildfire chronosequence. Global Change Biology, 10(4), 473-487.

Bradford, J. B., Lauenroth, W. K., & Burke, I. C. (2005). The impact of cropping on primary production in the US Great Plains. Ecology, 86(7), 1863-1872.

Campioli, M., Michelsen, A., Demey, A., Vermeulen, A., Samson, R., & Lemeur, R. (2009). Net primary production and carbon stocks for subarctic mesic–dry tundras with contrasting microtopography, altitude, and dominant species. Ecosystems, 12(5), 760-776.

Chen, W., Chen, J. M., Price, D. T., & Cihlar, J. (2002). Effects of stand age on net primary productivity of boreal black spruce forests in Ontario, Canada. Canadian Journal of Forest Research, 32(5), 833-842.

Del Grosso, S., Parton, W., Stohlgren, T., Zheng, D., Bachelet, D., Prince, S., ... & Olson, R. (2008). Global potential net primary production predicted from vegetation class, precipitation, and temperature. Ecology, 89(8), 2117-2126.

Doughty, C. E., Metcalfe, D. B., da Costa, M. C., de Oliveira, A. A., Neto, G. F. C., Silva, J. A., ... & Halladay, K. (2014). The production, allocation and cycling of carbon in a forest on fertile terra preta soil in eastern Amazonia compared with a forest on adjacent infertile soil. Plant Ecology & Diversity, 7(1-2), 41-53.

Fisk, M. C., Schmidt, S. K., & Seastedt, T. R. (1998). Topographic patterns of above-and belowground production and nitrogen cycling in alpine tundra. Ecology, 79(7), 2253-2266.

Frank, D. A. (2007). Drought effects on above-and belowground production of a grazed temperate grassland ecosystem. Oecologia, 152(1), 131-139.

Gao, Y. Z., Chen, Q., Lin, S., Giese, M., & Brueck, H. (2011). Resource manipulation effects on net primary production, biomass allocation and rain-use efficiency of two semiarid grassland sites in Inner Mongolia, China. Oecologia, 165(4), 855-864.

Gao, Y. Z., Giese, M., Lin, S., Sattelmacher, B., Zhao, Y., & Brueck, H. (2008). Belowground net primary productivity and biomass allocation of a grassland in Inner Mongolia is affected by grazing intensity. Plant and Soil, 307(1-2), 41-50.

Gill, R. A., Kelly, R. H., Parton, W. J., Day, K. A., Jackson, R. B., Morgan, J. A., ... & Zhang, X. S. (2002). Using simple environmental variables to estimate below-ground productivity in grasslands. Global ecology and biogeography, 11(1), 79-86.

Gilmanov, T. G., & Oechel, W. C. (1995). New estimates of organic matter reserves and net primary productivity of the North American tundra ecosystems. Journal of Biogeography, 723-741.

Girardin, C. A. J., Malhi, Y., Aragao, L. E. O. C., Mamani, M., Huaraca Huasco, W., Durand, L., ... & Salinas, N. (2010). Net primary productivity allocation and cycling of carbon along a tropical forest elevational transect in the Peruvian Andes. Global Change Biology, 16(12), 3176-3192.

Girardin, C. A., Espejob, J. E. S., Doughty, C. E., Huasco, W. H., Metcalfe, D. B., Durand-Baca, L., ... & Halladay, K. (2014). Productivity and carbon allocation in a tropical montane cloud forest in the Peruvian Andes. Plant Ecology & Diversity, 7(1-2), 107-123.

Gower, S. T., Krankina, O., Olson, R. J., Apps, M., Linder, S., & Wang, C. (2001). Net primary production and carbon allocation patterns of boreal forest ecosystems. Ecological applications, 11(5), 1395-1411.

Gower, S. T., Vogel, J. G., Norman, J. M., Kucharik, C. J., Steele, S. J., & Stow, T. K. (1997). Carbon distribution and aboveground net primary production in aspen, jack pine, and black spruce stands in Saskatchewan and Manitoba, Canada. Journal of Geophysical Research: Atmospheres, 102(D24), 29029-29041.

Helmisaari, H. S., Makkonen, K., Kellomäki, S., Valtonen, E., & Mälkönen, E. (2002). Below- and above-ground biomass, production and nitrogen use in Scots pine stands in eastern Finland. Forest Ecology and Management, 165(1-3), 317-326.

Herbert, D. A., & Fownes, J. H. (1999). Forest productivity and efficiency of resource use across a chronosequence of tropical montane soils. Ecosystems, 2(3), 242-254.

Hertel, D., Moser, G., Culmsee, H., Erasmi, S., Horna, V., Schuldt, B., & Leuschner, C. (2009). Below-and above-ground biomass and net primary production in a paleotropical natural forest (Sulawesi, Indonesia) as compared to neotropical forests. Forest Ecology and Management, 258(9), 1904-1912.

Huasco, W. H., Girardin, C. A., Doughty, C. E., Metcalfe, D. B., Baca, L. D., Silva-Espejo, J. E., ... & Huaraca-Quispe, L. P. (2014). Seasonal production, allocation and cycling of carbon in two mid-elevation tropical montane forest plots in the Peruvian Andes. Plant Ecology & Diversity, 7(1-2), 125-142.

Kajimoto, T., Matsuura, Y., Sofronov, M. A., Volokitina, A. V., Mori, S., Osawa, A., & Abaimov, A. P. (1999). Above-and belowground biomass and net primary productivity of a Larix gmelinii stand near Tura, central Siberia. Tree physiology, 19(12), 815-822.

Kalyn, A. L., & Van Rees, K. C. J. (2006). Contribution of fine roots to ecosystem biomass and net primary production in black spruce, aspen, and jack pine forests in Saskatchewan. Agricultural and Forest Meteorology, 140(1-4), 236-243.

Li, J., Lin, S., Taube, F., Pan, Q., & Dittert, K. (2011). Above and belowground net primary productivity of grassland influenced by supplemental water and nitrogen in Inner Mongolia. Plant and Soil, 340(1-2), 253-264.

Li, Z., Apps, M. J., Banfield, E., & Kurz, W. A. (2002). Estimating net primary production of forests in the Canadian Prairie Provinces using an inventory-based carbon budget model. Canadian Journal of Forest Research, 32(1), 161-169.

Litton, C. M., Raich, J. W., & Ryan, M. G. (2007). Carbon allocation in forest ecosystems. Global Change Biology, 13(10), 2089-2109.

Malhi, Y., Aragao, L. E. O., Metcalfe, D. B., Paiva, R., Quesada, C. A., Almeida, S., ... & Hutyra, L. R. (2009). Comprehensive assessment of carbon productivity, allocation and storage in three Amazonian forests. Global Change Biology, 15(5), 1255-1274.

Malhi, Y., Doughty, C. E., Goldsmith, G. R., Metcalfe, D. B., Girardin, C. A., Marthews, T. R., ... & da Costa, A. C. (2015). The linkages between photosynthesis, productivity, growth and biomass in lowland Amazonian forests. Global Change Biology, 21(6), 2283-2295.

Malhi, Y., Farfán Amézquita, F., Doughty, C. E., Silva-Espejo, J. E., Girardin, C. A., Metcalfe, D. B., ... & Marthews, T. R. (2014). The productivity, metabolism and carbon cycle of two lowland tropical forest plots in south-western Amazonia, Peru. Plant Ecology & Diversity, 7(1-2), 85-105.

Malhi, Y., Girardin, C. A., Goldsmith, G. R., Doughty, C. E., Salinas, N., Metcalfe, D. B., ... & Aragão, L. E. (2017). The variation of productivity and its allocation along a tropical elevation gradient: a whole carbon budget perspective. New Phytologist, 214(3), 1019-1032.

Martinez, C., Alberti, G., Cotrufo, M. F., Magnani, F., Zanotelli, D., Camin, F., ... & Rodeghiero, M. (2016). Belowground carbon allocation patterns as determined by the in-growth soil core 13C technique across different ecosystem types. Geoderma, 263, 140-150.

Martinez-Yrizar, A., Maass, J. M., Pérez-Jiménez, L. A., & Sarukhán, J. (1996). Net primary productivity of a tropical deciduous forest ecosystem in western Mexico. Journal of Tropical Ecology, 12(1), 169-175.

Moore, S., Adu-Bredu, S., Duah-Gyamfi, A., Addo-Danso, S. D., Ibrahim, F., Mbou, A. T., ... & Owusu-Afriyie, K. (2018). Forest biomass, productivity and carbon cycling along a rainfall gradient in West Africa. Global change biology, 24(2), e496-e510.

Moser, G., Leuschner, C., Hertel, D., Graefe, S., Soethe, N., & Iost, S. (2011). Elevation effects on the carbon budget of tropical mountain forests (S Ecuador): the role of the belowground compartment. Global Change Biology, 17(6), 2211-2226.

Newman, G. S., Arthur, M. A., & Muller, R. N. (2006). Above-and belowground net primary production in a temperate mixed deciduous forest. Ecosystems, 9(3), 317-329.

Ni, J. (2004). Estimating net primary productivity of grasslands from field biomass measurements in temperate northern China. Plant Ecology, 174(2), 217-234.

Ostertag, R. (2001). Effects of nitrogen and phosphorus availability on fine-root dynamics in Hawaiian montane forests. Ecology, 82(2), 485-499.

Prince, S. D., Haskett, J., Steininger, M., Strand, H., & Wright, R. (2001). Net primary production of US Midwest croplands from agricultural harvest yield data. Ecological Applications, 11(4), 1194-1205.

Ram, J., Singh, J. S., & Singh, S. P. (1989). Plant biomass, species diversity and net primary production in a central Himalayan high altitude grassland. The Journal of Ecology, 456-468.

Riutta, T., Malhi, Y., Kho, L. K., Marthews, T. R., Huaraca Huasco, W., Khoo, M., ... & Burslem, D. F. (2018). Logging disturbance shifts net primary productivity and its allocation in Bornean tropical forests. Global change biology.

Ruess, R. W., Cleve, K. V., Yarie, J., & Viereck, L. A. (1996). Contributions of fine root production and turnover to the carbon and nitrogen cycling in taiga forests of the Alaskan interior. Canadian journal of forest research, 26(8), 1326-1336.

Ryan, M. G., Lavigne, M. B., & Gower, S. T. (1997). Annual carbon cost of autotrophic respiration in boreal forest ecosystems in relation to species and climate. Journal of Geophysical Research: Atmospheres, 102(D24), 28871-28883.

Sierra, C. A., Harmon, M. E., Moreno, F. H., Orrego, S. A., & Del Valle, J. I. (2007). Spatial and temporal variability of net ecosystem production in a tropical forest: testing the hypothesis of a significant carbon sink. Global Change Biology, 13(4), 838-853.

Steele, S. J., Gower, S. T., Vogel, J. G., & Norman, J. M. (1997). Root mass, net primary production and turnover in aspen, jack pine and black spruce forests in Saskatchewan and Manitoba, Canada. Tree physiology, 17(8-9), 577-587.

Swamy, S. L., Dutt, C. B. S., Murthy, M. S. R., Mishra, A., & Bargali, S. S. (2010). Floristics and dry matter dynamics of tropical wet evergreen forests of Western Ghats, India. Current science, 99(3), 353-364.

Tan, Z., Zhang, Y., Yu, G., Sha, L., Tang, J., Deng, X., & Song, Q. (2010). Carbon balance of a primary tropical seasonal rain forest. Journal of Geophysical Research: Atmospheres, 115(D4).

Tateno, R., Hishi, T., & Takeda, H. (2004). Above-and belowground biomass and net primary production in a cool-temperate deciduous forest in relation to topographical changes in soil nitrogen. Forest Ecology and Management, 193(3), 297-306.

Van Do, T., Sato, T., Saito, S., & Kozan, O. (2015). Fine-root production and litterfall: main contributions to net primary production in an old-growth evergreen broad-leaved forest in southwestern Japan. Ecological research, 30(5), 921-930.

Vogt, K. A., Vogt, D. J., Palmiotto, P. A., Boon, P., O'Hara, J., & Asbjornsen, H. (1995). Review of root dynamics in forest ecosystems grouped by climate, climatic forest type and species. Plant and soil, 187(2), 159-219.

Wang, C., Gower, S. T., Wang, Y., Zhao, H., Yan, P., & Bond-Lamberty, B. P. (2001). The influence of fire on carbon distribution and net primary production of boreal Larix gmelinii forests in north-eastern China. Global Change Biology, 7(6), 719-730.

Xiao, C. W., Yuste, J. C., Janssens, I. A., Roskams, P., Nachtergale, L., Carrara, A., ... & Ceulemans, R. (2003). Above-and belowground biomass and net primary production in a 73-year-old Scots pine forest. Tree Physiology, 23(8), 505-516.

Xu, X., Niu, S., Sherry, R. A., Zhou, X., Zhou, J., & Luo, Y. (2012). Interannual variability in responses of belowground net primary productivity (NPP) and NPP partitioning to long-term warming and clipping in a tallgrass prairie. Global Change Biology, 18(5), 1648-1656.

Yang, Y. S., Chen, G. S., Guo, J. F., Xie, J. S., & Wang, X. G. (2007). Soil respiration and carbon balance in a subtropical native forest and two managed plantations. Plant Ecology, 193(1), 71-84.

Yuste, J. C., Konôpka, B., Janssens, I. A., Coenen, K., Xiao, C. W., & Ceulemans, R. (2005). Contrasting net primary productivity and carbon distribution between neighboring stands of Quercus robur and Pinus sylvestris. Tree Physiology, 25(6), 701-712.

Zhang, F., Quan, Q., Song, B., Sun, J., Chen, Y., Zhou, Q., & Niu, S. (2017). Net primary productivity and its partitioning in response to precipitation gradient in an alpine meadow. Scientific reports, 7(1), 15193.
