# Supplementary material for: Global subsoil organic carbon turnover times dominantly controlled by soil properties rather than climate
Source: Nat Commun. 2019 Aug 15;10:3688. doi: 10.1038/s41467-019-11597-9 (PMC6695437; doi:10.1038/s41467-019-11597-9)
Supplement: Supplementary file 3 — Description of Additional Supplementary Files [file 41467_2019_11597_MOESM3_ESM.pdf]

Description of Additional Supplementary Files

File name: Supplementary Data 1
Description: The fraction of NPP allocated to the 0.3 – 1 m soil layer at different locations across the globe.

File name: Supplementary Data 2
Description: Soil profile root biomass distribution.
