# Supplementary material for: Global subsoil organic carbon turnover times dominantly controlled by soil properties rather than climate
Source: Nat Commun. 2019 Aug 15;10:3688. doi: 10.1038/s41467-019-11597-9 (PMC6695437; doi:10.1038/s41467-019-11597-9)
Supplement: Supplementary file 2 — Peer Review File [file 41467_2019_11597_MOESM2_ESM.pdf]

Reviewers' comments:

Reviewer #1 (Remarks to the Author):

This paper addresses an important problem in cell biology: the mechanisms by which the spindle assembly checkpoint (SAC) alters the substrate selectivity of the APC/C, the ubiquitin ligase that initiates chromosome segregation in anaphase. In the presence of chromosomes that are not correctly aligned on the spindle, the SAC triggers formation of a protein complex (the MCC) that binds the APC/C and inhibits its activity toward some substrates (securin, cyclin B) but not others (cyclin A2). Past work has provided numerous clues about the multiple degron sequences and other mechanisms that contribute to this selectivity. In the current paper, the authors use a blend of biochemistry, cell biology, and structural biology to provide interesting new insights into this problem. Most importantly, they identify a previously unnoticed APC/C degron on cyclin A2, and they show that this degron cooperates with previously known degrons to promote APC/C-mediated ubiquitination of cyclin A2 in the absence and presence of the MCC. A major strength of the paper, which distinguishes it from other work in the field, is that the conclusions are supported by structural studies that provide intriguing insights into underlying mechanisms.

Concerns:

1. In Figure 6a, b, size exclusion chromatography is used to support the authors' claim that the Cdk2-cyclin A-Cks2 complex binds to APC/C-MCC without displacing the MCC. This conclusion is not well supported by the protein gel analysis. The amount of kinase complex associated with APC/C-MCC is clearly substoichiometric, and the kinase complex is dissociating during the column run. Thus, displacement of MCC would be difficult to quantify by simply looking at the MCC gel bands in the complex or in other fractions. Ideally, it would be helpful to see the results from parallel runs of APC/C-MCC or MCC on this column. The authors should soften their language in their interpretation of these results.

2. The results in Figure 7c and d are disappointing. Much of the paper is devoted to the importance of the D2 degron, and the lack of a clear effect when transplanted to cyclin B1 is a rather unfortunate way to end the paper. Given that this negative result could be due to a variety of uninteresting explanations, it could be argued that these results could be removed, allowing the paper to end on the strong points raised in Fig 7a and b.

Minor points:

1. I was initially confused by the authors' use of ubiquitination assays in which the reaction products are analyzed by western blotting with anti-ubiquitin, rather than by the more conventional analysis of the substrate. It might be helpful to summarize the method briefly in the legends.

2. On page 4-5, the text states: "a peptide modelled on residues 60-80 potently suppressed ubiquitination". In fact, inhibition seems to occur at tens or hundreds of micromolar peptide concentration, which I would not call potent. I would expect a good degron to have affinities in the 1-10 uM range.

3. The cartoons in Fig 4g-I are not ideal. In the D1 mode, the Cdc20 shift is not indicated, and it looks like D2 is bound to Cdc20.

4. Fig 7b: the legend does not make it clear that we are looking at a speculative model of how cyclin A2 binds, based on the previous APC-MCC structure.

Reviewer #2 (Remarks to the Author):

In their manuscript, Zhang et al. aim to shed new light on the mechanisms that control the ubiquitination of cyclin A2 by the APC/C to promote its early degradation in prometaphase, overcoming the SAC-dependent suppression of the APC/C activity towards other targets that are degraded later in the cell cycle, like cyclin B1. The authors identified a new non-canonical D box (D2) in cyclin A2 and evaluated the role of this degron sequence and how does it act in concert with the other three previously described degrons in this protein (a canonical D box (D1), a KEN box and an ABBA motif) to facilitate its recognition by the APC/C and to modulate the activity of this ubiquitin ligase towards cyclin A2. Specifically, the results in their manuscript show that mutation of the D2 box in cyclin A2 results in significantly reduced levels of the APC/C-dependent ubiquitination of this cyclin, which indicates that the D2 sequence is critical for its degradation. Furthermore, the D2 box, together with the KEN box and ABBA motif, allow cyclin A2 to overcome the inhibition of the APC/C activity by the SAC and be ubiquitinated by the APC/C in its MCC-repressed state. Finally, Zhang et al. postulate that cyclin A2 can associate with APC/C^{Cdc20} in either a high activity binding mode, mediated by the D2 and KEN boxes, or a low activity mode via the ABBA motif and D1 and KEN boxes. Their results uncover interesting new aspects of the regulation of the activity of APC/C towards its different substrates and make the manuscript potentially suitable for its publication in Nature Communications. However, and although the experiments described by the authors are mostly well executed and presented, important controls are missing in some cases. On the other hand, there are some observations that necessitate clarification and conclusions that need to be strengthened with additional experimentation. Hence, I consider that the manuscript is still too preliminary in its present form and would require further experimental support to grant its final publication.
My main concerns about the results are the following:
1.- The text is often confusing, especially when referring to repression of the APC/C complex activity by the MCC. As a representative example, in the sentence: “Our results reveal that the resistance of cyclin A2 to MCC inhibition is conferred by cooperative binding of four factors…” (page 10), one could mistakenly think that it is the MCC that is inhibited, and not that MCC inhibition of the APC/C is prevented. I would recommend to modify the manuscript, rephrasing the text in order to clarify all the instances where it could lead to this type of confusion.
2.- In most cases, the ubiquitination assays lack a negative control to ensure that the reaction is indeed APC/C-dependent, and therefore no ubiquitinated forms are observed in the absence of this ubiquitin ligase. Also, total levels of non-ubiquitinated Cyclin A2 or B1, and not only ubiquitinated forms starting from Ub1, should be displayed.
3.- Even though cyclin A2 is an early APC/C^{Cdc20} substrate, Supplementary Fig. 1a and 1b indicate that it is more efficiently and heavily ubiquitinated by APC/C^{Cdh1}. Could the authors comment about this observation?
4.- It would help to incorporate in all ubiquitination experiments some kind of quantification, similar to that shown in Fig. 7d, that helped with evaluating the extent to which the different substrates are modified by the APC/C.
5.- In Supplementary Fig. 1d it is stated that APC/C^{MCC} efficiently ubiquitinates cyclin A2 when is added at a concentration 33 times higher than that of the APC/C. How does this translate in terms of the intracellular concentration of the proteins? In general, it would be easier to interpret the results if the approximate relative intracellular concentration of Cyclin A2, APC/C, Cdc20, Cdh1 or MCC were provided.
6.- The authors postulate that the ABBA motif of cyclin A2 displaces the weaker second ABBA motif (A2) of BubR1, thereby associating with Cdc20. I wonder whether it would be possible to switch the ABBA motives of cyclin A2 and BubR1. If their model for how Cdk2-cyclinA2-Cks2 can be ubiquitinated by the MCC-repressed APC/C is correct, the prediction would be that, having a weaker ABBA motif, now cyclin A2 could not displace BubR1 and Cdk2-cyclinA2-Cks2 would be thus resistant to APC/C^{MCC} degradation.
7.- The authors should more extensively speculate about the possible physiological roles of the two binding modes of cyclin A2 with APC/C^{Cdc20}, since this observation could turn out to be just the result of an artificial conformation that is favored in the absence of the D2 box but

with no function whatsoever in the regulation of the activity of Cdk2-cyclinA2-Cks2.
8.- There is a general lack of statistical analyses (see Fig. 6d, 6f, 7d or Supplementary Fig. 2b, 2d, 2e), which are necessary to evaluate the significance of the differences shown between the different conditions that are compared.
Finally, some minor points are:
1.- In page 4, line 119, the initial sentence reads: “Previously findings…” [sic], instead of “Previous findings…”.
2.- In page 10, lines 310-311, the sentence: “… how cyclin A2 overcomes MCC inhibition and the roles individual degrons in cyclin A2 play…“ [sic] should be corrected (“…the roles that individual degrons…”).

Reviewer #3 (Remarks to the Author):

Zhang, Tischer, and Barford present an interesting paper building on Barford lab expertise on structural biochemistry of APC/C, coupled with Tischer’s in imaging in cell biology, to address the longstanding question of how Cyclin A2 is ubiquitylated/degraded during the spindle assembly checkpoint when other APC/C substrates are not. The authors take a systematic approach that leads to reporting a new, additional D-box “D2” sequence (previously recognized due to harbouring a Val replacement for the canonical Arg in the RxxLxD sequence). The authors show that this D2-box is crucial for Cyclin A2 recruitment to APC/C in some contexts. In addition to the identification of this Cyclin A2 degron, the paper has several major conclusions of broad significance:

- The authors show that two different degrons (here two different D-boxes, a KEN-box, an ABBA motif and presumably CKS protein binding to phosphorylated APC3 (although this is not experimentally addressed, it is implied) can be combined differently to achieve different substrate orientations on APC/C.

- Different degrons can contribute differently to substrate recruitment to APC/C activated by CDH1 and CDC20, and different rates of substrate degradation in cells.

- Cyclin A2’s ability to bind APC/C MCC depends on its strong ABBA motif competing off a weak ABBA motif from MCC, which along with D-box binding allows binding to CDC20M and ubiquitylation in the context of an APC/C-CDC20-MCC complex.

- Cryo EM structures of APC/C-CDC20-Cyclin A2-CDK2-CKS2 complexes could be interpreted in light of the mutagenesis to explain potential arrangement of substrate degrons.

- Highlights of these structures include that CDC20 can be positioned slightly differently positions during ubiquitylation of different substrates.

Overall, these are significant and important contributions to our understanding of cell cycle regulation, the spindle assembly checkpoint, and of this fascinating molecular machine, APC/C. The experiments are high quality and thorough, and should be published in Nature Communications. While the authors have made a tremendous systematic effort, the system is complex, leading to some issues that should be addressed, at least through changes to the text, prior to publication.

1. The order of the presentation is a little confusing, because the authors first define the degrons and through mutations of most of them individually and together determine their roles, then determine some structures, then perform comprehensive analysis of the degron roles and then determine another structure. I recognize this order is probably first to define the D2-box, then see structures, then analyse the structures, but overall the presentation becomes somewhat confusing

to the extent that only the most intrepid reader interested in APC/C will make it through to the end. While the mitosis and APC/C field are big enough to make this a pretty broad group, the work is really nice and could be appreciated by an even broader audience if the order of presentation were improved. The authors can figure out for themselves what they prefer, but I might recommend putting all the biochemistry up-front. This suggests that multiple substrate orientations on APC/C may be possible. Then the structural work.

2. In reading the structural interpretations, the fact that the structures can actually only be interpreted in light of the mutational analysis should come before the analysis. For example, page 6 line 175 seems to me can only be concluded AFTER the subsequent paragraph starting at line 179. So I think something along the lines of that there were two major classes that are interpreted based on the mutations should be stated up-front before the description of the structures. This is especially true of the density for the complex with APC/C-CDC20-MCC, which to me looks in the figures identical to density for complexes without Cyclin A2-Cdk2-CKS2.

3. The authors should find a way to overlay closeups of the relevant regions of the new EM maps with each other and with previous maps to show the actual differences in the comparisons. It is important for the uneducated reader to be able to visualize whether or not there are obvious differences in the EM density, and the extremely major extent to which interpretation of the maps relies on knowledge obtained from the biochemistry. This is especially true for the complex with MCC, where I surmise that the only experimental evidence for presence of Cyclin A2-CDK2-CKS2 in the complex is the difference in the ratios of MCC open versus MCC closed conformations.

4. I may have missed this, but how can the authors exclude potential differences in phosphorylation accounting for the different conformations. I was not sure what construct was used to express CDK2. Many laboratories coexpress CDK2 with a CDK-activating enzyme, which would render the Cyclin A2-CDK2-CKS2 complex an active kinase. If active CDK2 was used, this needs to be clarified early in the text as a potential contributing factor to differences (although this reviewer does agree that the major differences in ubiquitylation pattern most likely result from degron differences). Even if non-CAK activated CDK2 was used, some weak activity of CDK2, in the context of high concentrations and a massive molar excess relative to the APC/C complexes could lead to some APC/C (or CDC20 or MCC) phosphorylation. Such phosphorylation would presumably depend on how Cyclin A2 is recruited to the different complexes (i.e., which spatial arrangements are possible – not just CKS2 recruitment to the APC3 loop in this case as is shown in the cartoon, but also recruitment of the Cyclin A2 degrons to CDC20 or CDC20M in the case of the MCC complex). This is especially true in light of the author's own data – that they cannot visualize a position for the globular kinase complex relative to the APC/C, implying that the flexibly tethered kinase active site could encounter potential substrates.

5. If the authors cannot rule out that the kinase has not changed the phosphorylation status of the APC/C-CDC20-MCC complex, do they have any additional data besides the change in ratio of open to closed conformation that the Cyclin A2-CDK2-CKS2 complex is actually associated with APC/C-CDC20-MCC in the particles visualized by cryo EM? If not, I don't think this precludes publication in Nature Communications with the data in-hand. However, the interpretation of the EM data becomes further muddied, and the authors need to simply state that they cannot absolutely determine if the density observed bound to MCC comes from the APC/C-CDC20-MCC itself or loosely tethered Cyclin A2-CDK2-CKS2, or whether the change in ratio of open/closed conformations comes from binding to Cyclin A2 degrons or from phosphorylation by this kinase.

6. Differences in phosphorylation may also contribute to the different degradation rates in cells. The cellular data are absolutely beautiful, but this potential contributing factor should just be stated. Although the rates of degradation of other substrates may be unaffected, there could be controls of phosphorylation at different stages of the cell cycle that correct for any effects of phosphorylation during the SAC.

Response to reviewers' comments

Reviewer #1 (Remarks to the Author):

This is an interesting manuscript that calculates the turnover time of subsoil C pools. More information on the behavior of this carbon would lead to a better understanding of the impacts of climate change on SOC stocks.

Response: We thank the reviewer very much for these encouraging comments. We have thoroughly revised the manuscript based on the comments. See the detailed point-by-point responses below.

I think there are two main problems with this manuscript. First, the biome-average numbers used seem unlikely to be very robust. The algorithm using the data from Jackson et al. (1996) is based on data that's now more than 20 years old. I suspect the reason that there hasn't been an update is that it's really difficult to measure belowground allocation. And I guess these are the most recent data. Even if these data were indicative of allocation patterns, the observation set for this database doesn't overlap with the data used to construct the WSRC database. It may be the case that each of these sources does a good job of representing each biomes (an aside: it would have been nice to see sample numbers and info on distributions w/in each biome). Even so, it's not clear how meaningful biome-wide data on soil turnover times are. The distribution (Table S3) suggests that differences between biomes may be insignificant.

Response: We greatly appreciate these comments on the turnover times among biomes. We agree with the point that measuring belowground carbon allocation is a significant challenge. Fortunately, Jackson et al. (1996) did an excellent work to estimate this allocation biome by biome via synthesizing global data sets. Their estimation is comprehensive and the performance of their approach in estimating belowground carbon allocation is robust, albeit this work had been done more than 20 years ago. Although the data is old, the credibility and value of their findings based on the data apparently do not fade with time and is still valid. In this study, we build upon their findings to infer belowground carbon allocation.

We also accept that the approach and the data used to estimate belowground carbon allocation are imperfect and have some limitations, mainly due to limited data availability resulting from the difficulty of direct measuring as highlighted above. We unfortunately cannot progress this and it is also beyond the scope of this study. However, we have elucidated the discussions on the potential effects of imperfect estimation of belowground C allocation, the mismatch of scale between HWSD database and C allocation estimations (all other databases used in this study are at the same resolution as HWSD including climate, NPP and global biome distribution), and other limitations. Please see lines 213-233. *PS: the reviewer mentioned the WSRC database, we are not sure about this database as we do not use it in our study and also do not*

know what the WSRC refer to. We assume that it refers to the HWSD database so we discussed the scale mismatch between HWSD and C allocation estimations.

We appreciate the comment on the distribution of turnover times among biomes and the potential differences. By considering this comment, we have produced another figure to clearly show the distribution of turnover times among the nine biome types (Fig. 2). In addition, we expanded the Table S3 to include the median of turnover times in the nine biome types, and the 97.5% and 2.5% quantiles are also included. Overall, Fig. 2 and Table S3 provide enough information on the difference of the distribution of turnover times among biomes. In general, the turnover times are shorter in wetter and/or hotter biomes, and *vice versa*. This result is meaningful, and consistent with current understanding and our assessment on the role of climate (Fig. 4). We have incorporated the relevant results in lines 86-94.

Second, the construction of the manuscript seems to suggest that by understanding the relative influence of the driers of equilibrium SOC stocks, we will understand the drivers of transient effects on SOC stocks. This may be true - long-term change could drive wholesale substitution of (e.g.,) tropical forests w/ tropical savannas. Also, several aspects of current SOC stocks are legacies of long-term change (e.g., the high C silt loams formed near the southern extent of glaciers 10k years ago). In fact, leading-edge soil science and the models that integrate it explicitly consider the factors that lead to formation and stabilization of SOC. I'm curious how an assessment of the relative impact of different SOC formation/loss drivers would map out in comparison with this study. That said, it is clear that the idea that multiple factors driver SOC formation and loss is already embedded in current SOC science. It may appear that they are not because the emphasis is on the drivers that're subject to change (primarily climate but also management and vegetation).

Response: Thank the reviewer for these detailed comments. This comment mainly relates to the novelty, scope and motivation of this study. To our knowledge, our study is the first to spatially-explicitly quantify SOC turnover times in world subsoils (i.e., the 0.3 – 1 m soil layer) at the resolution of ~1 km across the globe. As we have concisely introduced in the first paragraph in the main text, our current understanding of the SOC stabilization and stabilization is mainly built upon the research of the top soil layers (< 0.3 m) at small scales (i.e., field experiments and plot-scale measurements). One reason for the limited research in the subsoil is that it is difficult to undestructively monitor subsoil SOC dynamics. Another reason would be that subsoil SOC is usually considered to be relatively stable compared with topsoil SOC, although several new studies (at the plot level, e.g., Pires et al. 2017) have challenged this view. Our study used the best-available observation-derived global information to estimate turnover times at the global scale. We found that subsoil SOC can be very active indicated by the fast turnover times in some regions (e.g., < 60 years in most southern American and African regions). Our new assessment on the distribution of turnover time among biomes (thank the

reviewer for this suggestion) indicates that turnover times are skewed to small values. Our estimation provides a benchmark for diagnosing subsoil SOC dynamics under climatic and land use change. Our estimation will be also particularly useful for verifying predictions of Earth system model – a dominate tool for predicting carbon cycle-climate feedbacks and guiding policy making. We have highlighted and explicitly discussed these points throughout the manuscript (e.g., in lines 116-123, 165-166, 245-251).

We have to emphasize that our study estimates the turnover times of SOC at the global scale as well as at the local scale, and identifies the drivers controlling the spatial variability at both scales. We have a breaking through finding that climate is secondary in influencing subsoil SOC turnover compared with the influence of soil properties. It is traditionally accepted that climate is the most important driver controlling SOC dynamics. Indeed, most soil carbon and Earth system models simulating SOC dynamics are fundamentally driven by climate. Our results demonstrated that this is not true for SOC in the subsoil. To further emphasize our novel findings and the relevant implications, we expanded the last conclusion paragraph (lines 245-251).

In addition, to satisfy the concerns on the legacy effects of other variables that have not been considered in this study, we expanded the relevant discussion in a paragraph highlighting the limitations of this study (lines 221-225).

In sum, this interesting piece of work. I believe it could be improved by going into more depth on distributions of SOC and NPP-soil within each biome, linking to observations that explicitly couple both (e.g., 14C data?), and discussing how mechanisms of SOC formation/stabilization/loss vary across biomes.

Response: Thank the reviewer very much for providing all the helpful and constructive comments. We have carefully considered all comments/suggestions. We have made the following key modifications relating to the above two major comments raised by this reviewer.

1. We draw a new figure (Fig. 2) to show the distribution of SOC, NPP and turnover times of SOC among the nine biomes to clearly show the difference of these variables among the nine biomes.
2. We have expanded the relevant discussions (e.g., lines 116-129, 165-166, 178-179, 191-194, 213-233) to highlight the novelty and implications of our results, and limitations.

We did not explicitly compare our observation-based estimates of turnover times with other data sources (e.g., 14C data mentioned by the reviewer). There are several reasons. First, although 14C data would be very useful for estimate SOC turnover time, the data is relatively scarce, particularly when comparing with our global scale high resolution estimates. Second, we estimate the apparent turnover time of SOC under the assumption of steady state. As we have highlighted in lines 56-61, 227-230 , strict steady

state is rare, and 14C data may not meet this steady state assumption. In addition, most 14C data were collected after the period of nuclear bomb test (i.e., 1950s-1960s). Indeed, the first research using 14C data to study SOC dynamics occurred in 1964 (Paul et al. 1964). So the relevant estimates may only reflect the turnover time during the period from the bomb-test period to the sampling time. More importantly, using 14C data to estimate SOC turnover has a series of uncertainties caused by the assumptions underlying the different approaches (see the detailed discussion by Bruun, Six and Paustian, 2005). Although we do not explicitly compare our estimation of turnover times with other data sources (largely due to that direct measurements of turnover time are unavailable), we have compared our results with other studies at the similar spatial scales (see lines 116-129).

References:

Bruun S, Six J, Jensen LS, Paustian K. 2005. Estimating turnover of soil organic carbon fractions based on radiocarbon measurements. Radiocarbon 47: 99-113.

Paul EA, Campbell CA, Rennie DA, McCallum KJ. 1964. Investigations of the dynamics of soil humus utilizing carbon dating techniques. Proceedings of the 8th International Congress of Soil Science 3:201–208.

Pries CEH, Castanha C, Porras R, Torn M. 2017. The whole-soil carbon flux in response to warming. Science 355: 1420-1423.

Reviewer #2 (Remarks to the Author):

In their manuscript “Global subsoil carbon turnover times and their drivers” by Z. Luo et al. present global distributions of the observation-based estimates of turnover times of the soil organic matter (SOM) and identify causes of variation in these estimates. The authors report that climatic variables (mean annual temperature and precipitation) account for 40% of spatial variation in the SOM turnover times, while soil properties, such as cation exchange capacity, acidity, bulk density, texture, etc. account for the other 60%. The authors also suggest using high-resolution turnover times and relative importance of climate and soil properties as a benchmark for evaluating management strategies.

Response: Thank the reviewer for summarizing our study.

Soils present a great challenge for the carbon cycle modelling community as evidenced by the poor model performance in representing soil carbon estimates (Todd-Brown et al., 2013). There is a need for insights into the mechanisms of soil carbon dynamics, and it was intriguing how this manuscript would address the issue. The authors used a machine learning technique and open-source data to tease apart the effects of climate and soil properties on SOM turnover

time, however it was not entirely convincing to me that (1) model was constructed in a meaningful way; (2) findings are useful for model development.

Response: Thank the reviewer very much for the insightful and constructive comments. Based on the comments/suggestions, we have thoroughly revised the manuscript. See our point-by-point response below.

The explanatory variables for the model included bulk density and cation exchange capacity (CEC). As authors point out in the methods section, bulk density is used to calculate SOM stocks from SOM concentrations, so it seems logical that bulk density would be correlated with SOM turnover times, because turnover times, in turn, are calculated from SOM stocks. CEC is associated with soil organic matter content, but is not a cause of high organic matter content: CEC increases with SOM content, because soil organic matter itself is negatively charged due to dissociation of organic acids. Since turnover times are calculated from SOM content, I don't think CEC should be included into the model as explanatory variable.

Response: Thank the reviewer very much for the careful review and the detail explanation on why BD and CEC should be excluded from the model. We followed these suggestions, and re-performed the model by excluding BD and CEC. The reanalysis indicated that excluding BD and CEC from the model does not significantly affect the model performance (Fig. 4). Using the updated BRT model, the model can explain 93% the variance in SOC turnover times. The relevant text and figures/tables have been updated based on the new results.

Overall, I suggest authors to place more emphasis on describing and interpreting the effects of soil properties on SOM turnover times in the next version of the manuscript. Such approach will be more useful for constructing new experiments and model development, and also make the variables without the meaningful explanatory values more evident.

Response: Thanks for these suggestions. In the revised version, we provided another paragraph to specifically discuss the effects of soil properties on SOC turnover (see lines 178-194). However, we have to note that, unlike the effect of climate, soil environment is complex involving numerous interactions between physical, chemical and biological properties. Our current understanding is not enough to identify specific mathematic forms to reflect the relevant effects of the complex soil matrix. As such, in the manuscript, we emphasized that "we need novel approaches to derive metrics representing soil heterogeneity that can generally describe the effects of various soil properties on SOC dynamics". In lines 245-251, we also highlight that a specific subsoil module is required in order to properly capture subsoil SOC dynamics, as prevailing carbon models are mainly developed based on knowledge of topsoil SOC dynamics and primarily driven by climate (which is conflict with the findings in this study).

The r-squared in the Figure 3b is truly striking. However, it was not clear to me whether it was calibration or validation fit. Although I am not very familiar with the boosted regression tree

(BRT) method, in my experience some classification and regression tree methods may result in over-fitting and validating the models on an independent data set is critical. Was BRT model validated on a random sample of pixels (that did not include the calibration pixels)? In lines 303-306 it looks like a validation procedure, and if it is, it needs to be more clearly stated here and in the figure caption.

Response: Thanks very much for these comments. Yes, the procedure of fitting the BRT model do include an explicit cross-validation process (10 fold cross-validation in this study), making the BRT robust to overfitting (Elith, Leathwick and Hastie, 2008). However, to satisfy the concern on the predictive performance of the BRT model and following the suggestion of the reviewer, we further validated the model on an independent data set (see Fig. 4). The model can explain 86% variance in the validation data set, demonstrating the robustness of our approach.

Regarding the identification of relative importance of climate and edaphic factors. If I understood correctly, the authors applied a boosted regression tree model to region 100x100 km (10,000 data points), and identified the relative importance of climate and edaphic factors. It seems to me that unless it's a mountainous area, climatic variables are not going to vary much in 100x100 km regions, so one would consistently detect a more substantial contribution from edaphic factors than from climatic factors, because SOM turnover times would likely be more variable than climatic variables (on a small scale). What if the sampling design is different, e.g. turnover times are analyzed in vertical swaths (i.e. swaths along the longitudes)? Would temperature have more weight under such sampling design?

Response: Thanks very much for the careful review and suggestions. For the assessment at the local scale, the reviewer's understanding is right. Fig. 3 shows the relevant results and indicates that edaphic factors are not always more important than climate, and the relative importance of climate is strongly location-dependent (It is important to note that Fig. 3A shows the distribution of the relative importance of climate based on the BRT analysis at 10,000 100x100 km regions across the global). The latitudinal pattern was also presented in Fig. 3B. On average, however, our results suggested climate is less important than soil properties. In addition, we presented some new results in Fig. S3 which shows the proportion of the variable that is the most important for subsoil SOC turnover at the local scale (~100×100 km) across the globe.

Except the relative importance of climate and edaphic factors at the local scale, we have another assessment at the global scale. At this scale, we randomly selected 10,000 pixels (1x1 km) to conduct the BRT analysis. The results were presented in Fig. 4. At the global scale, our conclusion was that climate is secondary in controlling the spatial variability of SOC turnover times compared with soil properties, but temperature indeed is the most important individual variable.

We did not further expand our assessment using other sampling designs such as along the longitudes and/or along the latitudes. The reason is that, at both global and local scales, it is clear and general from our results that soil properties are more important than climate in regulating SOC turnover although some variability at the local scale. At the global scale, temperature is the important individual variable. At the local scale, we produced another figure (Fig. S3) to show the most important individual variables at each of the 10,000 100x100 km regions. We believe this new figure can clarify the concern on the relative importance of temperature mentioned in the comment.

Once the expressed issues are addressed, I think the paper will be an important contribution to understanding SOM dynamics.

Response: Thanks again for the insightful and constructive comments. We have addressed all issues point-by-point as specified above. We believe the reviewer will be more satisfied by this new version.

Below, please see the minor comments: Line 36: "The turnover time of SOC, which can be estimated as the residence time of a carbon atom in the soil" If by "residence time of carbon atom" you meant radiocarbon estimates, I'd suggest not comparing the two. Radiocarbon estimates are the estimates that account for non-steady state, and the estimates in the manuscript are the steady-state estimates.

Response: Thanks for this suggestion. In the revision, we have simply deleted "*which can be estimated as the residence time of a carbon atom in the soil*", which does not affect the meaning of the sentence.

Line 85-86: It is interesting why turnover times in the croplands are higher than temperate grasslands or temperate forests. In the manuscript it is suggested that croplands are transformed temperate forests, which means that the higher turnover times in croplands are either due to higher SOM or lower belowground NPP in croplands than in temperate forests. Since cropland SOM is likely lower than temperate forest SOM, do differences in NPP's account for the differences in the turnover times between croplands and temperate forests?

Response: Thanks for the careful review and the suggestion on the potential difference in NPP between croplands and temperate biomes. By considering this suggestion, we have compared the NPP between cropland and temperate forests and grassland in Fig. 2C. The result suggests that average NPP in world croplands is moderate compared with the NPP in temperate forests and grasslands.

However, we have expanded the relevant discussions in lines 100-115. Crops usually have shallower rooting depth than native forest or grassland, and thus allocate less photosynthesized C to the subsoil (i.e., NPP allocated to the subsoil). This would be another reason for the estimated longer turnover time of C in cropland subsoils.

Another reason is that subsoil SOC in croplands is not at the steady state. Please see the detailed discussions in lines 100-115.

Line 94-95: Comparisons to references #11 and 18 are invalid, because in (11) turnover time calculated using top 1 m SOM and total NPP, as opposed to subsoil SOM and belowground NPP in this study; and in reference #18 the turnover times are obtained with topsoil SOM.

Response: Thanks for the careful review. We revised the relevant comparisons by specifying the soil depth in each study and changed the relevant context. See lines 116-123.

Line 125: “climate dominance” is not sufficiently addressed in the manuscript. What information should it convey to the reader?

Response: Thanks for this comment. In this study, we discarded the usage of “climate dominance”, and converted to a more general expression of “climate is more important than soil properties” or similar. The whole manuscript has been updated on this.

Line 146: Could one of the reasons be overfitting by the BRT?

Response: Thanks for this comment. I have further validate the model performance using an independent data set. See our detailed response to a comment above.

Line 181: “guild”; did you mean “guide”?

Line 275: correct “MAT” to “MAP”

Response: Thanks for these careful review. Changed accordingly.

Todd-Brown KEO, Randerson JT, Post WM, Hoffman FM, Tarnocai C, Schuur EaG, Allison SD (2013) Causes of variation in soil carbon simulations from CMIP5 Earth system models and comparison with observations. Biogeosciences, 10, 1717-1736.

Response: This reference has been cited in the manuscript with the relevant discussion on modelling using Earth system models.

Reviewers' comments:

Reviewer #1 (Remarks to the Author):

This is my second review of this manuscript. Overall the authors did a nice job of responding to comments on the initial version. That said, turnover is indirectly proportional to NPP allocation to subsurface soils and uncertainty in this term is not assessed in any way. My interpretation of their methods is that they used the distribution of NPPsoil derived from interannual variability in total NPP as a surrogate for uncertainty. This is not really uncertainty - it's year-on-year variability. Taking a time-integrated average of this is not unreasonable (if this variability represents the longer-term variability).

However, this approach fails to account for the uncertainty w/in their data on NPP allocation belowground and to subsoil, which I believe is probably very large. The authors state that "We unfortunately cannot progress this and it is also beyond the scope of this study." I would argue that because this analysis is so heavily dependent on this below 0.3 C inputs, that taking the time to review this literature and properly incorporate that uncertainty into their analysis is essential.

Reviewer #2 (Remarks to the Author):

After carefully going through reviewers' comments and authors' responses, I'd like to recommend another round of revision.

First, the authors dismissed the suggestion made by one of the reviewers to compare their residence time estimates with the radiocarbon-based age estimates. In my view radiocarbon-based age estimates should not be dismissed, because they provide an important validation dataset for steady state assumption. I suggest authors include the suggested comparison into their manuscript. The radiocarbon-based age estimates can be found here: https://powellcenter-soilcarbon.github.io/soilcarbon/

Second, it is very confusing why after removing two variables (CEC and bulk density) that explained a substantial amount of variation in residence time, the r-squared for the calibration data set did not decrease, but became even higher than it was. That phenomenon was not addressed by authors in the response letter, and it would be interesting to find out the underlying reason for no decrease in r-squared. Can it be that explanatory variables are correlated? If that is the case, perhaps the authors should reduce the dimensionality of the model?

Third, I suggest the authors lay out the logical flow more carefully, as it was hard to follow in some places (e.g. paragraph in lines 95-115).

Lastly, I suggest revising the grammar (e.g. lines 18, 26, 182, 234, etc).

Note: All major revisions in the manuscript are marked as BLUE colour. Taking advantage of the less format restriction of Nature Communications, in addition to address the reviewers' comments, we also reorganised the manuscript including adding subsections and separating results, discussion and conclusions. We believe this kind of re-organization can enhance the readability of the paper and can make the finding clearer for the reader.

Reviewer #1 (Remarks to the Author):

This is my second review of this manuscript. Overall the authors did a nice job of responding to comments on the initial version. That said, turnover is indirectly proportional to NPP allocation to subsurface soils and uncertainty in this term is not assessed in any way. My interpretation of their methods is that they used the distribution of NPPsoil derived from interannual variability in total NPP as a surrogate for uncertainty. This is not really uncertainty - it's year-on-year variability. Taking a time-integrated average of this is not unreasonable (if this variability represents the longer-term variability).

Response: We thank reviewer #1 for her/his positive assessment on our revision and for the constructive suggestions on how to further improve our work. Based on the comments on uncertainty, we have thoroughly revised the manuscript based on new data sets, and have explicitly quantified the uncertainty induced by NPP allocation belowground and to subsoil. Details see below our response to the next comment.

In addition, we also considered the uncertainty in estimating average NPP. For total NPP, ideally, we need long-term average NPP. However, we only have the NPP data from 2001 to 2015. A simple way is to use the average of the 15 yr NPP data, but this ignores the potential uncertainty in total NPP estimation induced by the limited 15 yr data. In this study we used a bootstrapping approach to quantify the uncertainty in average NPP estimated with the 15 yr NPP data.

However, this approach fails to account for the uncertainty w/in their data on NPP allocation belowground and to subsoil, which I believe is probably very large. The authors state that "We unfortunately cannot progress this and it is also beyond the scope of this study." I would argue that because this analysis is so heavily dependent on this below 0.3 C inputs, that taking the time to review this literature and properly incorporate that uncertainty into their analysis is essential.

Response: Thank the Reviewer #1 very much for the constructive suggestion on incorporating potential uncertainty sources into the estimation of SOC turnover times. In this revision, we have conducted a comprehensive literature search and screened hundreds of published papers to compile a dataset of belowground NPP allocation. We believe our dataset on the belowground NPP allocation is the most comprehensive one to date, and this data set has been provided as the Supplementary Database 1. After derived the belowground NPP application, we further used the root biomass distribution dataset obtained from ORNL DAAC (https://daac.ornl.gov/cgi-bin/dsviewer.pl?ds_id=660) to infer the fraction of belowground NPP

that is allocated to the 0.3–1 m soil layer (Supplementary Database 2). These observational datasets were used in the revision to estimate uncertainty induced by NPP allocation belowground and to subsoil, and the whole section on the estimation of turnover times (please see the revised sections "*The allocation of NPP*" and "*Estimation of SOC turnover times and its uncertainty*") and all relevant presentations (including all figures/tables and relevant descriptions) have been revised.

Reviewer #2 (Remarks to the Author):

After carefully going through reviewers' comments and authors' responses, I'd like to recommend another round of revision.

Response: Thank the reviewer very much for her/his careful review and providing the insightful comments.

First, the authors dismissed the suggestion made by one of the reviewers to compare their residence time estimates with the radiocarbon-based age estimates. In my view radiocarbon-based age estimates should not be dismissed, because they provide an important validation dataset for steady state assumption. I suggest authors include the suggested comparison into their manuscript. The radiocarbon-based age estimates can be found here: https://powellcenter-soilcarbon.github.io/soilcarbon/

Response: We appreciate Reviewer #2's suggestion on the comparison between the estimated turnover times (τ) and radiocarbon age. Reviewer #1 had the same suggestion during the first round of review. Using the recommended radiocarbon data, we have estimated radiocarbon age in 535 soil profiles using a common model for estimating carbon age in soils based on the ratio of ^{14}C to ^{12}C of SOC (the detailed model was provided independently as a supplementary information for review only). We compared the estimated τ using Equation (4) in the manuscript with that estimated by radiocarbon data at the global and biome level (which is done by grouping the 535 profiles into the nine biomes). The following figure shows the result.

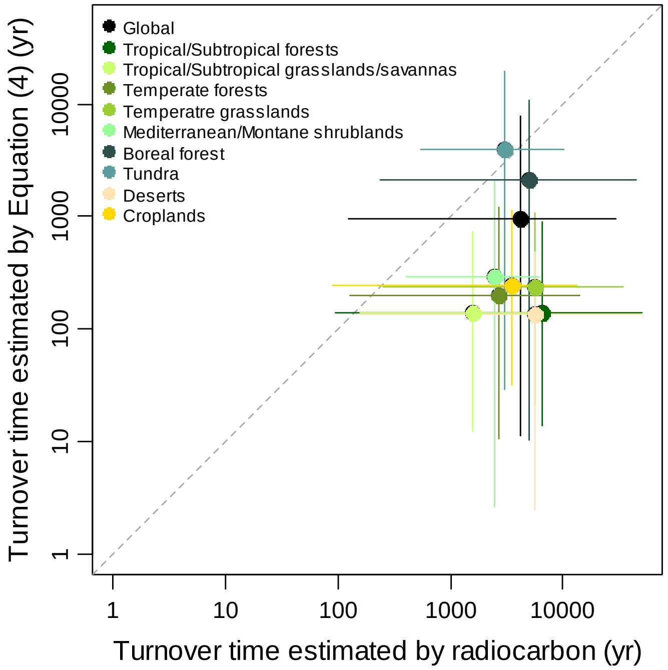

In this figure, the dot points show the average and the bars show the range (i.e., 5% and 95% quantiles), while the dashed line shows the 1:1 line. The results indicate that the global average of radiocarbon-estimated τ is 4,242 yr ranging from 122 (5% quantile) to 29,551 (95% quantile) years. Although the relative ranges of τ estimated from both methods are similar, radiocarbon-estimated global average τ is four times higher than the Equation (4)-estimated τ. Among the nine biomes, except the two cold biomes – tundra and boreal forest, radiocarbon-estimated τ is also much higher than the Equation (4)-estimated τ for the other seven biomes. The potential reasons for this discrepancy of estimated τ using the two approaches are multi-folds.

First, a big problem in such comparisons is the large differences in underlying assumptions. In the radiocarbon dataset, only the total SOC was reported, and thus the radiocarbon model has to assume a homogeneous SOC pool with its turnover described by first-order kinetics. In reality, however, SOC is not homogeneous, but consists of carbon components with different degradability. Another piece of important information required by the radiocarbon model to estimate τ is the ^{14}C content of the plant materials entering the soil, but this information is also not known in the data set. We have to indirectly infer it using the atmospheric ^{14}C content at the time of plant growth. In addition, for all soil profiles in the data set, only one measurement of soil ^{14}C content is reported. Using this one measurement, we cannot test the steady state assumption. Rather the radiocarbon model itself has to assume an equilibrium between input and decay. For these reasons, the radiocarbon model may significantly over- or under-estimate turnover times. The approach presented in the manuscript is also not perfect, but with some limitations which have been specified in the *Limitations* section in the revised manuscript.

Second, the sampled soil layers in different soil profiles are different in the radiocarbon data set. Particularly, a large fraction of the soil profiles does not include continuous soil layers, some soil layers are missed, and some soil profiles are only sampled to a depth less than 1 m. In our comparison, we did not exclude those soil profiles as it will substantially reduce the sample size. These limitations of the radiocarbon dataset make it impossible to obtain unbiased estimation of radiocarbon age in the 0.3 – 1 m soil profile (which is the focus of our estimation).

Third, our study is at the global scale, while ^{14}C data is measured at a specific location. Although we can extract the estimated τ in a typical pixel (at the resolution of 1 km), this τ is not based on NPP allocation to subsoil *in situ*, while we considered NPP allocation as an uncertainty source (please see our response to Reviewer #1's comment on the uncertainty sources). By looking into the details of the ^{14}C data, we found that in some locations, multiple soil profiles are measured but the reported ^{14}C values are very different. This leads to very different radiocarbon age. Our global assessment cannot capture such spatial variability at the fine scale.

After all, because of the assumptions underlying the radiocarbon age estimation, the limitations of the radiocarbon data and our approach, and the mismatch of scales, the comparison may turn this into an "apples vs oranges" kind of comparisons. We sincerely appreciate Reviewer #2's suggestion on the comparison with radiocarbon age (Reviewer #1 also had required this). After assessing the radiocarbon data and careful thinking, at last, we determined to not include this comparison in this particular study. We believe this kind of comparison may be more valid when the data used under different methodology frameworks are collected at the same scale and have the same data quality.

Second, it is very confusing why after removing two variables (CEC and bulk density) that explained a substantial amount of variation in residence time, the r-squared for the calibration data set did not decrease, but became even higher than it was. That phenomenon was not addressed by authors in the response letter, and it would be interesting to find out the underlying reason for no decrease in r-squared. Can it be that explanatory variables are correlated? If that is the case, perhaps the authors should reduce the dimensionality of the model?

Response: Thank the reviewer for the careful review and highlighting the increased r^2 (from 0.92 to 0.93) after removing CEC and BD from the machine learning model. A reason for the increased r^2 (from 0.92 to 0.93) would be that we randomly selected 10,000 data points to fit the BRT model. As such, the fitted r^2 may be a little bit different for each model-fitting. Another reason would be that CEC and/or BD may be largely correlated to some other predictors in the model. For this case, if we remove CEC and BD, the quality of the fit would only change little as the information of CEC and BD can be represented by other predictors (because of the correlation). In addition, the BRT will find other nonlinearities and/or interactions to compensate for the loss of predictive power due to the removal of CEC and BD.

Thank the reviewer on the suggestion of reducing the dimensionality of the model. A big advantage of machine learning model is its ability to model high-dimensional data set, taking into account nonlinearities and interactions. In addition, the machine learning model can quantify the relative importance of each individual variable. These features of machine learning have been summarized in lines 446–451. If we reduce the dimensionality of the model (e.g., using a generalized variance as an indicator of the overall variation of several predictors), we will loss the information of the relative importance which is a major question addressed in this study.

Third, I suggest the authors lay out the logical flow more carefully, as it was hard to follow in some places (e.g. paragraph in lines 95-115).

Lastly, I suggest revising the grammar (e.g. lines 18, 26, 182, 234, etc).

Response: Thank the reviewer very much for the careful review and the above two comments. We have carefully checked the whole manuscript for potential logical and grammar issues, and revised correspondingly to make the manuscript clearer for the reader.

Reviewers' comments:

Reviewer #2 (Remarks to the Author):

The authors did a great job evaluating and incorporating reviewers' suggestions regarding uncertainty quantification and comparison of modelled residence times with the residence times derived from C isotope data. In my view, the manuscript has improved greatly.

I still have reservations, however, regarding the BRT technique and what we can and cannot learn from it. I think that to draw conclusions about what exactly influences SOC residence times, we need to remove correlated predictors. In the first version of the manuscript CEC and BD had a substantial effect on SOC residence times, but after dropping those predictors from the model, no predictive power was lost. Authors argue for not reducing dimensionality of the model, because the information on the relative importance of predictors might be lost, but it seems to me that if eight soil variables can be represented with, e.g., three, the total effect of soil properties on SOC residence time would remain the same. Also authors state that "the BRT will find other nonlinearities and/or interactions to compensate for the loss of predictive power due to the removal of CEC and BD", which makes me wonder, what if all soil variables are removed? How much variance will be explained with MAT and MAP only? Previous exercise with removing BD and CEC showed that it may not necessarily be 44% as Fig. 5a suggests…

I hope I was able to convey my uncertainty about this approach. At this point I'd recommend contacting an additional reviewer with expertise in BRT application to evaluate the conclusions made based on this method.

Lastly, below are some minor suggestions:
L45-46: "Although several studies have estimated ecosystem carbon turnover times at the global scale,…"
L 101-103 This piece of information does not seem to be elaborated on in discussion, so it is not clear why the focus is drawn to it here.
L185: "In this study we quantified spatially explicit turnover times…"
L189: In reference 13 the SOC turnover time is calculated as the ratio of total SOC for top 1 m to total NPP (as opposed to belowground NPP). The values in that study don't seem to be comparable to the residence times in this study.
L300-301: this finding does not align with the experiment shown in Supplementary Figure 4a. Why?
L336-337: please revise, some text seems to be missing

Reviewer #3 (Remarks to the Author):

BRT Application :

Overall, the fitting and evaluation (validation) of BRT seems, from the provided information, correct and relevant bibliographic references are given. The response to reviewer #2 is convincing regarding BRT application. However, some specific comments may be raised
• In the methods section, it would be interesting to get the parameters used to fit BRT models (tree depth, number of individual trees and so on). Optimal parameters may change from one location to another (for the 10,000 BRT models experiment) and one has to make sure that each BRT model was fit using the right parameters.
• The authors write that "Although the relative and overall importance of climate and soil can be quantified by the BRT analysis, another important question is the direction of the effect of each variable. To address this question, we performed a path analysis". Some readers may understand from this sentence that BRT cannot be used to address the direction of the effect of each variable,

when it actually does, thanks to partial dependence plots. The authors should clarify this and maybe justify why they did not use this buit-in BRT tool and preferred the (otherwise interesting) path analysis.
• I don't think the way relative variable importance (RI) are aggregated over the 10,000 BRT models is adequate, because it might indeed mix very different things : let us say we have two BRT model, one m1 with a good fit and predictive performance (say R2 = 0.8) and another one, m2, with a very poor fit and predictive performance (say R2 = 0.05). Even for the poorly fitted BRT model, m2, we may estimate a variable importance for a given variable v1 (say with a score of RI=80). For m1, let us say that RI=10. If you now take the average RI for both models, as it is done in the paper over 10,000 models, to assess the importance of V1 for predicting the target property, you will find that it is 45, which is not true since one should trust more m1 and not trust m2, and rather conclude that, where one was able to reasonably model the target variable, importance of v1 was low. Thus maybe, prior to averaging RIs, the author should rather find a way to weight RIs based on models predictive performance. Even when looking at RIs plotted against latitude (fig. 4.B), we don't really know if all values are equally trustable (meaning come from models with similar predictive performance).

Statistical modeling of carbon turnover times and uncertainty analysis :

• I was actually very surprised by the very good validation statistic of the BRT model (fig. 5), which is indeed very close to the training statistics. Usually, when mapping soil properties, especially when dealing with subsoil, model performance on a validation dataset is much lower, among other because of high spatial variability of soil properties. Then I recalled that in the present study, the models do not attempt to predict real turn over rates but merely indirectly estimated ones (as mentioned in fig.5 caption l. 651) from the HWSD database and Modis NPP. One explanation for such a good predictive performance might be that in HWSD, SOC levels are inherently correlated to other soil properties and broad climatic zones and these correlations define the soil units. In turn, doesn't the trained BRT model simply models how SOC levels were defined by HWSD authors, and how, together with the other soil variables and climatic variables they were used to defined broad soil types? It might not be surprising that a model using HWSD soil parameters and climate covariates is well able to predict a variable, carbon turnover time, which primarily relies on HWSD SOC estimates. Remaining unexplained variability might simply result from the NPP variability. This should be tested by fitting the BRT models with the same covariates on SOC and on NPP separately. One would then check whether the very good performance in predicting estimated turn over (SOC/NPP) comes from an accurate modeling of HWSD SOC levels or of Modis NPPs (which per se would be very interesting and could help discussing NPP controls l.225-231), or none (which would demonstrate that working on SOC/NPP is really an added value). In turn, if the BRT model is merely a reproduction of HWSD SOC levels (with variability within soil units coming from Modis NPP data), the BRT models could maybe be advantageously replaced by a simpler analysis taking into account soil classes instead basic soil properties. To summarize, if the BRT very good fit simply results from the intrinsic HWSD correlations between soil properties and climate characteristics on one side, and SOC on the other side, subsequent developments based on this BRT model regarding carbon turnover controls have limited interest.
• I agree with Reviewer #1 that inter-annual NPP variability is not really NPP uncertainty. It is true that variance of the NPP mean within the time period can be approached using bootstrapping. However, this is not equivalent to the uncertainty resulting from the Modis algorithm, for each year or averaged over a time period. This later variance is the one to be considered in the uncertainty analysis. We should note, though, that it was interesting to include the uncertainty of the other BNPP(0.3-1) parameters (eq.3) in the analysis. However, the authors should go further and derive the contribution of each component (assuming a proper variance for the NPP estimate can be provided). I am not specialist for deriving contributions with a monte-carlo experiment set-up, and cannot recommend a specific method but see Saltelli et al. (2008) for specific methods.
• I disagree with the authors about the negligible contribution of SOC levels to carbon turnover time variations, simply based on the fact that carbon turnover time ranges from several decades to centuries (l.397). Errors in SOC spatial predictions are sometimes important. For instance, for

France, Soilgrid products (Hengl et al. 2017) over-estimate by 100% SOC stock below 30cm (Mulder et al. 2016). If SOC is over-estimated by 100% so will be the carbon turnover time, and hence uncertainty related to SOC estimates is not negligible. Tifafi et al. (2018) also compared global SOC products and found great discrepancies, depending on the pedo-climatic zones, such as the boreal zones where HWSD SOC estimates rely on very few soil observations. This should be taken into account when drawing conclusions about such areas (e.g. l.256). l. 101-103 authors claim that fig. 2 demonstrate the weak influence of SOC. This is not the conclusion I have when looking at it and maybe simply changing the y axis range for fig. 2B (excluding SOC extreme values) would change the conclusion of the authors regarding this point. For all these reasons, I suggest that a proper uncertainty analysis of carbon turnover time should also include uncertainty on SOC. One could for instance use several available SOC maps as input or include directly uncertainty assessment available for some of these soil maps (for instance, globalsoilmap products or soilgrid do include uncertainty of their SOC estimates). Not considering it should at least be acknowledged as a possible limitation of the present study.

Using the model of SOC turnover times for making SOC projections :

I was not comfortable with this approach, for several reasons :
1. Does it make sense to increase homogeneously temperature by 2 degrees? I would rather use spatial RCP scenarios, in order to take into account possible correlations between temperature change and SOC or NPP spatial distributions.
2. HWSD is known to be based on few observations and possibly inaccurate for high latitude zones (Tifafi et al. 2018, Batjes et al. 2009, Carvalhais et al. 2014) where SOC might actually be extremely sensitive to warming. Hence, the proposed global estimate might be biased.
3. Isn't it obvious that since temperature change on carbon turnover time is almost neutral here, increasing NPP by 10% in SOC = (carbon turnover time * NPP) will yield a 10% increase in SOC (l.172-173)? Maybe did I misunderstood something here.
4. It seems cumbersome to first model carbon turnover time based on SOC and NPP estimates, then to multiply carbon turnover time modeled values by NPP estimates used in a first place to fit the model, to eventually get back to SOC estimates. If the goal is to make SOC projections, modelling (statistically) SOC directly (possibly using NPP estimates as covariates) would have been more straightforward and maybe discussion about relevant drivers, uncertainty of projections would have been more straightforward too. See for instance Meersmans et al. 2016 or Yigini et al. 2016.

Minor remarks and questions:
l.35 : please give a reference or more detail. I do not see why, although more difficult, manipulation below 30cm is more destructive.
l.211 : supplementary Fig. 2. is about Latitudinal patterns of SOC turnover times
l.276 : soil depth and qualification of rooting conditions are available in HWSD. Was it considered in the present study for adjusting the root biomass distribution in the soil profile

Meersmans, J., Arrouays, D., Rompaey, A.J.J.V., Pagé, C., Baets, S.D., Quine, T.A., 2016. Future C loss in mid-latitude mineral soils: climate change exceeds land use mitigation potential in France. Scientific Reports 6, 35798. https://doi.org/10.1038/srep35798
Yigini, Y., Panagos, P., 2016. Assessment of soil organic carbon stocks under future climate and land cover changes in Europe. Sci. Total Environ. 557, 838–850.
Hengl, T., de Jesus, J.M., Heuvelink, G.B.M., Gonzalez, M.R., Kilibarda, M., Blagotic, A., Shangguan, W., Wright, M.N., Geng, X., Bauer-Marschallinger, B., Guevara, M.A., Vargas, R., MacMillan, R.A., Batjes, N.H., Leenaars, J.G.B., Ribeiro, E., Wheeler, I., Mantel, S., Kempen, B., 2017. SoilGrids250m: Global gridded soil information based on machine learning. PLoS One 12, e0169748.
Mulder, V.L., Lacoste, M., Richer-de-Forges, A.C., Martin, M.P., Arrouays, D., 2016. National versus global modelling the 3D distribution of soil organic carbon in mainland France. Geoderma 263, 16–34.

Tifafi, M., Guenet, B., Hatte, C., 2018. Large Differences in Global and Regional Total Soil Carbon Stock Estimates Based on SoilGrids, HWSD, and NCSCD: Intercomparison and Evaluation Based on Field Data From USA, England, Wales, and France. Glob. Biogeochem. Cycle 32, 42–56.
Batjes, N.H., 2009. Harmonized soil profile data for applications at global and continental scales: updates to the WISE database. Soil Use and Management 25, : 124–127. https://doi.org/10.1111/j.1475-2743.2009.00202.x

Response to reviewers' comments

We are grateful for the reviewers for their positive comments on our last round revision and their constructive and helpful suggestions to further improve the manuscript. Their comments have helped us to think more deeply about certain aspects of our work. As a result, our manuscript has benefited and largely improved. We have revised the MS taking into account all comments raised. If we disagreed with a comment, we explain why and provide our perspective with evidence from new data analysis and/or the literature, when possible. We also reformatted the MS according to the format requirements by *Nature Communications*. Below are our detailed responses (in BLUE text) to each of the reviewer's comments (in BLACK text).

Reviewers' comments:

Reviewer #2 (Remarks to the Author):

The authors did a great job evaluating and incorporating reviewers' suggestions regarding uncertainty quantification and comparison of modelled residence times with the residence times derived from C isotope data. In my view, the manuscript has improved greatly.

Response: Thank the reviewer very much for the positive comments.

I still have reservations, however, regarding the BRT technique and what we can and cannot learn from it. I think that to draw conclusions about what exactly influences SOC residence times, we need to remove correlated predictors. In the first version of the manuscript CEC and BD had a substantial effect on SOC residence times, but after dropping those predictors from the model, no predictive power was lost. Authors argue for not reducing dimensionality of the model, because the information on the relative importance of predictors might be lost, but it seems to me that if eight soil variables can be represented with, e.g., three, the total effect of soil properties on SOC residence time would remain the same. Also authors state that "the BRT will find other nonlinearities and/or interactions to compensate for the loss of predictive power due to the removal of CEC and BD", which makes me wonder, what if all soil variables are removed? How much variance will be explained with MAT and MAP only? Previous exercise with removing BD and CEC showed that it may not necessarily be 44% as Fig. 5a suggests…

I hope I was able to convey my uncertainty about this approach. At this point I'd recommend contacting an additional reviewer with expertise in BRT application to evaluate the conclusions made based on this method.

Response: We appreciate these comments on the BRT application. Regarding to the questions raised here, we fitted another BRT model driven by MAT and MAP only (Fig. 1) as suggested by the review. The results suggest that the model can explain 48% of the variance. Indeed, this result is very close to the expectation of 44%. The 4% discrepancy (48% - 44%) is acceptable considering the potential difference in modelled interactions and nonlinear relationships by BRT excluding and including soil variables. We also tested the BRT performance using MAT, MAP, and the three most important soil variables as drivers (Fig. 2). By including the three variables, the percentage of explained variance is increased to 87%. These examples demonstrate the

validity of our BRT application. Please refer to our detailed response to reviewer 3's comments on the BRT application.

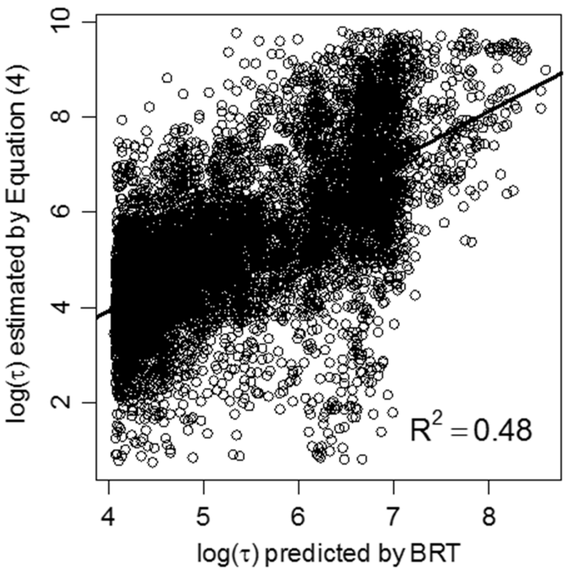

Figure 1. The performance of the BRT driven by MAT and MAP only in predicting estimated τ.

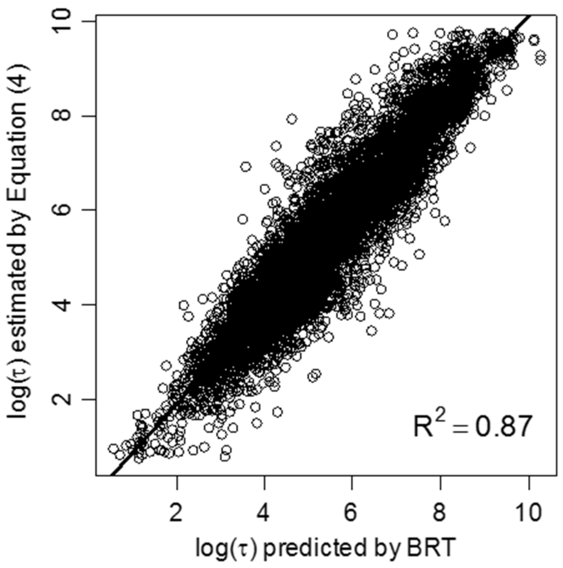

Figure 2. The performance of the BRT driven by MAT, MAP, and the three most important three soil variables shown in the manuscript.

Lastly, below are some minor suggestions:

L45-46: "Although several studies have estimated ecosystem carbon turnover times at the global scale,…"

Response: Changed accordingly.

L 101-103 This piece of information does not seem to be elaborated on in discussion, so it is not clear why the focus is drawn to it here.

Response: Thanks for the careful review. We deleted this piece of information.

L185: “In this study we quantified spatially explicit turnover times…”

Response: Changed accordingly.

L189: In reference 13 the SOC turnover time is calculated as the ratio of total SOC for top 1 m to total NPP (as opposed to belowground NPP). The values in that study don’t seem to be comparable to the residence times in this study.

Response: We have revised the relevant sentence to make it clearer. The main purpose of citing the mentioned reference is to highlight that SOC turnover times in different soil layers should be estimated separately, and NPP allocation also should be considered.

L300-301: this finding does not align with the experiment shown in Supplementary Figure 4a. Why?

Response: Thanks for the careful review. Supplementary Fig. 4a in the last version manuscript shows the distribution of the response of SOC to warming. Indeed, SOC change under 2°C warming ranges from <–50% to >80%, demonstrating the sensitivity of SOC to warming, albeit the global average change is neutral. To avoid potential ambiguity, we changed “vulnerability” to “sensitivity” in the mentioned sentence. In addition, we have deleted the whole section on the model experiments of the response to warming. See our responses to Reviewer 3’s comments.

L336-337: please revise, some text seems to be missing

Response: Thanks for the careful review. Revised.

Reviewer #3 (Remarks to the Author):

BRT Application :

Overall, the fitting and evaluation (validation) of BRT seems, from the provided information, correct and relevant bibliographic references are given. The response to reviewer #2 is convincing regarding BRT application.

Response: Thank the reviewer very much for these comments and acknowledging that our previous responses to reviewer #2 is convincing regarding the application of BRT.

However, some specific comments may be raised

Response: We appreciate these comments. Below, we have addressed all of them.

• In the methods section, it would be interesting to get the parameters used to fit BRT models (tree depth, number of individual trees and so on). Optimal parameters may change from one location to another (for the 10,000 BRT models experiment) and one has to make sure that each BRT model was fit using the right parameters.

Response: Yes, each BRT model of the 10,000 modelling experiments has its own optimal parameters, but it is possible (it would be also not necessary as it is too technical for most readers) to list all parameters considering page limitations. Alternatively, we have provided the relevant R scripts fitting BRT models to which interested readers can refer.

• The authors write that "Although the relative and overall importance of climate and soil can be quantified by the BRT analysis, another important question is the direction of the effect of each variable. To address this question, we performed a path analysis". Some readers may understand from this sentence that BRT cannot be used to address the direction of the effect of each variable, when it actually does, thanks to partial dependence plots. The authors should clarify this and maybe justify why they did not use this buit-in BRT tool and preferred the (otherwise interesting) path analysis.

Response: Thanks for this suggestion. Except the direction of the effect, path analysis has an advantage of detecting indirect and direct effects (e.g., how climate affects turnover times via its effect on soil), which is the main reason we prefer this method. We have clarified this in the revised MS (see lines 438-440).

• I don't think the way relative variable importance (RI) are aggregated over the 10,000 BRT models is adequate, because it might indeed mix very different things: let us say we have two BRT model, one m1 with a good fit and predictive performance (say R2 = 0.8) and another one, m2, with a very poor fit and predictive performance (say R2 = 0.05). Even for the poorly fitted BRT model, m2, we may estimate a variable importance for a given variable v1 (say with a score of RI=80). For m1, let us say that RI=10. If you now take the average RI for both models, as it is done in the paper over 10,000 models, to assess the importance of V1 for predicting the target property, you will find that it is 45, which is not true since one should trust more m1 and not trust m2, and rather conclude that, where one was able to reasonably model the target variable, importance of v1 was low. Thus maybe, prior to averaging RIs, the author should rather find a way to weight RIs based on models predictive performance. Even when looking at RIs plotted against latitude (fig. 4.B), we don't really know if all values are equally trustable (meaning come from models with similar predictive performance).

Response: Thanks for this suggestion and we understand the concerns made by the reviewer. We think there are some misunderstandings here. It must be pointed out that the referred importance is the RELATIVE IMPORTANCE, which is independent of the predictive performance of the model. Following on the example of the reviewer, m1 has a good performance with a R^2 of 0.8, which means soil and climatic variables can explain 80% of the variation of SOC turnover times. If soil contributes 50% to the explained variables (i.e., 80%), climate will contributed the left 50%. For m2, even though it has a poor performance with a R^2 of 0.05, if soil contributes 50% to the explained variables (i.e., 5%), climate will still contribute the left 50%. **That is, the model**

predictive performance does not affect the RELATIVE IMPORTANCE of soil or climate. To address the concerns on the different performance of the 10,000 BRT models, we have generated another figure to show the distribution and latitudinal pattern of their R^2 (Supplementary Fig. 4) and discus the relevant model performance (lines 125-132).

Statistical modeling of carbon turnover times and uncertainty analysis :

• I was actually very surprised by the very good validation statistic of the BRT model (fig. 5), which is indeed very close to the training statistics. Usually, when mapping soil properties, especially when dealing with subsoil, model performance on a validation dataset is much lower, among other because of high spatial variability of soil properties. Then I recalled that in the present study, the models do not attempt to predict real turn over rates but merely indirectly estimated ones (as mentioned in fig.5 caption l. 651) from the HWSD database and Modis NPP. One explanation for such a good predictive performance might be that in HWSD, SOC levels are inherently correlated to other soil properties and broad climatic zones and these correlations define the soil units. In turn, doesn't the trained BRT model simply models how SOC levels were defined by HWSD authors, and how, together with the other soil variables and climatic variables they were used to defined broad soil types?

It might not be surprising that a model using HWSD soil parameters and climate covariates is well able to predict a variable, carbon turnover time, which primarily relies on HWSD SOC estimates. Remaining unexplained variability might simply result from the NPP variability. This should be tested by fitting the BRT models with the same covariates on SOC and on NPP separately. One would then check whether the very good performance in predicting estimated turn over (SOC/NPP) comes from an accurate modeling of HWSD SOC levels or of Modis NPPs (which per se would be very interesting and could help discussing NPP controls l.225-231), or none (which would demonstrate that working on SOC/NPP is really an added value). In turn, if the BRT model is merely a reproduction of HWSD SOC levels (with variability within soil units coming from Modis NPP data), the BRT models could maybe be advantageously replaced by a simpler analysis taking into account soil classes instead basic soil properties.

To summarize, if the BRT very good fit simply results from the intrinsic HWSD correlations between soil properties and climate characteristics on one side, and SOC on the other side, subsequent developments based on this BRT model regarding carbon turnover controls have limited interest.

Response: Thank the reviewer for the careful review and detailed suggestions on how to check the intrinsic HWSD correlations. For "the very good validation" results, it should be emphasized that we used the **10-fold cross-validation** strategy as described in the MS to derive the BRT. Then, the BRT was further validated using another independent data set (grey circles in Fig. 5b). Thus, it is not surprising to see the very close performance of the BRT model in simulating the two datasets.

To clarify whether SOC and other soil properties in the HWSD are estimated based on their own/inherent correlations, we have carefully read the publications on the Harmonized World Soil Database (http://www.fao.org/docrep/018/aq361e/aq361e.pdf). In brief, soil units in

the HWSD are defined based on different soil classification systems (e.g., FAO-90 units) which are independent of soil physiochemical properties (which are based on measurements of thousands of soil profiles across the globe). Based on the pre-defined soil units and soil profile measurements, SOC and other soil properties across the globe at the resolution of 1 km were derived using taxonomy-based pedotransfer rules. Here, we want to highlight that inherent correlations among soil properties may exist. We can conduct a correlation analysis to detect potential correlations as we have done here via BRT analysis. However, this correlation does not invalidate our assessment focusing on turnover times of SOC (i.e., SOC/NPP) as long as the relevant data is independent. This is also true for NPP. Following the suggestion made by the reviewer, we fitted models for both SOC (Fig. 3) and NPP (Fig. 4). The results indicated that both SOC and NPP can be well explained by BRT driving by the same variables for SOC turnover times. A piece of interesting finding is that the variable importance is different for SOC (Fig. 3), NPP (Fig. 4) and SOC/NPP (Fig. 5 in the main manuscript), which also demonstrates the value of focusing on SOC/NPP.

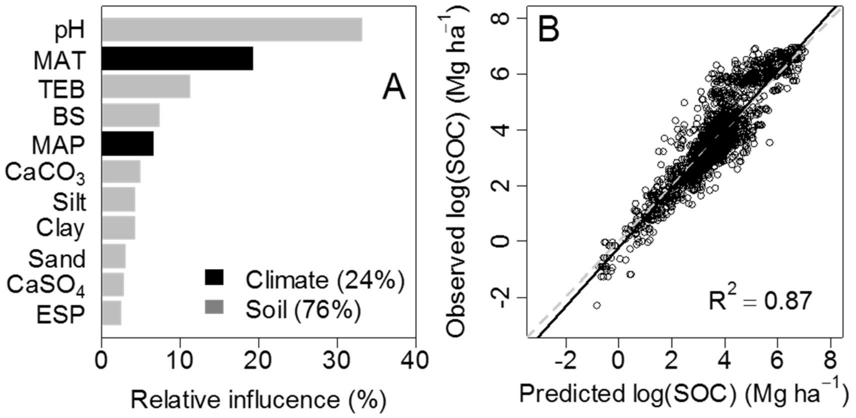

Figure 3. The relative influence of factors affecting subsoil SOC stock (A) and the performance of BRT in simulating subsoil SOC stock (B) using the variables shown in panel A.

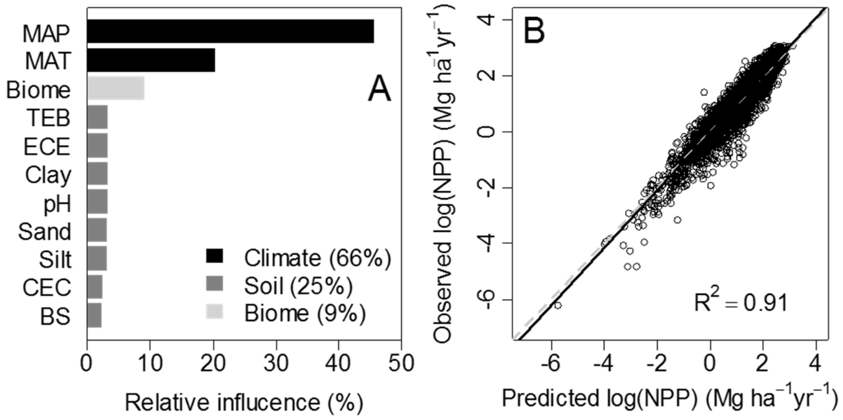

Figure 4. The relative influence of factors affecting NPP (A) and the performance of BRT in simulating NPP (B) using the variables shown in panel A.

We do think the results presented Figs. 3 and 4 themselves are very interesting for understanding controls over the spatial pattern of SOC and NPP, but we do not want this assessment overwhelms our assessment on SOC turnover times which are the focus of this study, a key variable indicating soil carbon stability and dynamics, as well as the key message the paper intends to communicate. Fortunately, if our manuscript finally can be accepted for publication by Nature Communications, our response to reviewers will be also available online for interested readers. For the relevant discussion on the effect of SOC and NPP, we have cited relevant references to support our statements and/or discussions.

• I agree with Reviewer #1 that inter-annual NPP variability is not really NPP uncertainty. It is true that variance of the NPP mean within the time period can be approached using bootstrapping. However, this is not equivalent to the uncertainty resulting from the Modis algorithm, for each year or averaged over a time period. This later variance is the one to be considered in the uncertainty analysis. We should note, though, that it was interesting to include the uncertainty of the other BNPP(0.3-1) parameters (eq.3) in the analysis. However, the authors should go further and derive the contribution of each component (assuming a proper variance for the NPP estimate can be provided). I am not specialist for deriving contributions with a monte-carlo experiment set-up, and cannot recommend a specific method but see Saltelli et al. (2008) for specific methods.

Response: We agree with the reviewer that the uncertainty in NPP is important for the estimation of SOC turnover times. There are mainly two uncertainty sources in the MODIS NPP product including upstream inputs (e.g., leaf area) of the algorithm for calculating NPP as well as the algorithm itself (Zhao et al., 2005). In this study, we used the improved MODIS NPP product, which has used the best-available input information and the state-of-the-art algorithm (Zhao et al., 2005. Improvements of the MODIS terrestrial gross and net primary production global data set. Remote sensing of Environment 95, 164-176). This NPP product is also the most comprehensive NPP dataset at the global scale and had been widely used in the relevant scientific community (e.g., Zhao et al., 2010. Drought-Induced Reduction in Global Terrestrial Net Primary Production from 2000 Through 2009. Science 329, 940-943). However, unfortunately, there is no concrete assessment to date on the uncertainty in this NPP product. The key challenge is the limited available field data in comparison to global coverage data sets, and the mismatch in scale between ground-based NPP measurements and the resolution (1 km) of the NPP product. In the revision, we have emphasized these limitations and challenges (lines 371-374).

Although the difficulty to explicitly quantify the uncertainty in the MODIS NPP product resulting from the algorithm and its inputs (which is a totally different research area. In this study, we used the NPP product to estimate SOC turnover times at the global scale), we used an empirical approach to estimate NPP uncertainty and propagate this uncertainty into the estimation of SOC turnover times. Specifically, we quantified the uncertainty in NPP in each 1 km pixel by assuming a normal distribution of NPP with a mean estimated by the 15-year MODIS NPP product and standard deviation (SD) assigned as the 10% of the mean, i.e., *NPP~N(mean, SD)*. Then, a 200 Monte Carlo simulations were conducted to estimate NPP

uncertainty by randomly sampling the normal distribution. Please see details on this approach in lines 374-379. Based on this uncertainty assessment, all figure and table presentations and the relevant descriptions in the whole manuscript have been updated.

• I disagree with the authors about the negligible contribution of SOC levels to carbon turnover time variations, simply based on the fact that carbon turnover time ranges from several decades to centuries (l.397). Errors in SOC spatial predictions are sometimes important. For instance, for France, Soilgrid products (Hengl et al. 2017) over-estimate by 100% SOC stock below 30cm (Mulder et al. 2016). If SOC is over-estimated by 100% so will be the carbon turnover time, and hence uncertainty related to SOC estimates is not negligible. Tifafi et al. (2018) also compared global SOC products and found great discrepancies, depending on the pedo-climatic zones, such as the boreal zones where HWSD SOC estimates rely on very few soil observations. This should be taken into account when drawing conclusions about such areas (e.g. l.256). l. 101-103 authors claim that fig. 2 demonstrate the weak influence of SOC. This is not the conclusion I have when looking at it and maybe simply changing
the y axis range for fig. 2B (excluding SOC extreme values) would change the conclusion of the authors regarding this point. For all these reasons, I suggest that a proper uncertainty analysis of carbon turnover time should also include uncertainty on SOC. One could for instance use several available SOC maps as input or include directly uncertainty assessment available for some of these soil maps (for instance, globalsoilmap products or soilgrid do include uncertainty of their SOC estimates). Not considering it should at least be acknowledged as a possible limitation of the present study.

Response: We appreciate the comments and understand the concern raised about the uncertainty of SOC. To identify the importance of SOC stock in estimating SOC turnover times, we have directly assessed the relationships of the estimated turnover times with subsoil SOC stock and with NPP (see Fig. 2 in the revised MS). The results indeed demonstrate the importance of SOC stocks. The original Fig. 2 was deleted from the MS as this figure largely repeated the information (e.g., the distribution of estimated turnover times) shown in Table 1.

For the uncertainty in SOC, we contend that the HWSD is the best choice for this typical study. The main reason is that the HWSD is consistent with other databases used in this study in terms of spatial coverage and resolution, eliminating potential uncertainties induced by resolution mismatching. Unlike soilgrid or other similar products like WISE30sec, more importantly, SOC and soil properties in the HWSD are independent of climate and land use (see our response above), which benefits the assessment on the controls. Otherwise, we will really encounter the "inherent correlation" issue as the reviewer commented above. For example, SoilGrids predict SOC and other soil properties considering climate and land use as covariates (Hengl et al., 2017). Although we did not explicitly quantify the effect of the uncertainty in SOC on the estimation of turnover times, following the suggestion of the reviewer, we have discussed the limitations of using the HWSD database by referring to the publications the reviewer suggested and others (see lines 243-247).

Using the model of SOC turnover times for making SOC projections:

I was not comfortable with this approach, for several reasons:

1. Does it make sense to increase homogeneously temperature by 2 degrees? I would rather use spatial RCP scenarios, in order to take into account possible correlations between temperature change and SOC or NPP spatial distributions.

2. HWSD is known to be based on few observations and possibly inaccurate for high latitude zones (Tifafi et al. 2018, Batjes et al. 2009, Carvalhais et al. 2014) where SOC might actually be extremely sensitive to warming. Hence, the proposed global estimate might be biased.

3. Isn't it obvious that since temperature change on carbon turnover time is almost neutral here, increasing NPP by 10% in SOC = (carbon turnover time * NPP) will yield a 10% increase in SOC (l.172-173)? Maybe did I misunderstood something here.

4. It seems cumbersome to first model carbon turnover time based on SOC and NPP estimates, then to multiply carbon turnover time modeled values by NPP estimates used in a first place to fit the model, to eventually get back to SOC estimates. If the goal is to make SOC projections, modelling (statistically) SOC directly (possibly using NPP estimates as covariates) would have been more straightforward and maybe discussion about relevant drivers, uncertainty of projections would have been more straightforward too. See for instance Meersmans et al. 2016 or Yigini et al. 2016.

Response: Thank the reviewer very much for raising these concerns and the detailed comments. We simply deleted the section on SOC projections. This deletion does not diminish the originality of the study's key findings on the global mapping of subsoil SOC turnover times and the underlying controlling factors. In addition, the message from these modelling experiments can be obtained via our BRT modelling results. Indeed, the results have demonstrated the importance of temperature, which is the most important factor controlling SOC turnover times at the global scale as well as at the local scale.

Minor remarks and questions:

l.35 : please give a reference or more detail. I do not see why, although more difficult, manipulation below 30cm is more destructive.

Response: Thanks for the careful review. The original sentence was revised to "*Main challenges are that subsoil SOC dynamics are more difficult to detect in situ*".

l.211 : supplementary Fig. 2. is about Latitudinal patterns of SOC turnover times

Response: Thanks for the careful review. It should be Table 2.

l.276 : soil depth and qualification of rooting conditions are available in HWSD. Was it considered in the present study for adjusting the root biomass distribution in the soil profile

Response: Thank the reviewer for pointing out this. Indeed, we also had noticed the information of soil depth and rooting conditions in the HWSD. However, the data is difficult to use in our study. For the soil depth in the HWSD, it is defined as the reference depth of the soil unit and is a constant value of 100 cm for the majority of soil units. For the rooting condition, the HWDS

provides the depth class of an obstacle to roots, which cannot reflect the real root distribution in the soil profile. In addition, this information is limited to the top 80 cm soil layer and only available for the European Soil Database (which is a sub-database of HWSD). These limitations inhibit explicit use of these two pieces of information in our application. Rather, we had synthesised a global database of measured root biomass distribution in the 0.3 – 1 m soil layer in different biomes and considered the uncertainty of root distribution in the estimation of turnover times in our last round revision. To explicit quantify the effect of soil depth and rooting conditions, we need novel approach to collect more comprehensive datasets as we have discussed in the manuscript.

Meersmans, J., Arrouays, D., Rompaey, A.J.J.V., Pagé, C., Baets, S.D., Quine, T.A., 2016. Future C loss in mid-latitude mineral soils: climate change exceeds land use mitigation potential in France. Scientific Reports 6, 35798. https://doi.org/10.1038/srep35798

Yigini, Y., Panagos, P., 2016. Assessment of soil organic carbon stocks under future climate and land cover changes in Europe. Sci. Total Environ. 557, 838–850.

Hengl, T., de Jesus, J.M., Heuvelink, G.B.M., Gonzalez, M.R., Kilibarda, M., Blagotic, A., Shangguan, W., Wright, M.N., Geng, X., Bauer-Marschallinger, B., Guevara, M.A., Vargas, R., MacMillan, R.A., Batjes, N.H., Leenaars, J.G.B., Ribeiro, E., Wheeler, I., Mantel, S., Kempen, B., 2017. SoilGrids250m: Global gridded soil information based on machine learning. PLoS One 12, e0169748.

Mulder, V.L., Lacoste, M., Richer-de-Forges, A.C., Martin, M.P., Arrouays, D., 2016. National versus global modelling the 3D distribution of soil organic carbon in mainland France. Geoderma 263, 16–34.

Tifafi, M., Guenet, B., Hatte, C., 2018. Large Differences in Global and Regional Total Soil Carbon Stock Estimates Based on SoilGrids, HWSD, and NCSCD: Intercomparison and Evaluation Based on Field Data From USA, England, Wales, and France. Glob. Biogeochem. Cycle 32, 42–56.

Batjes, N.H., 2009. Harmonized soil profile data for applications at global and continental scales: updates to the WISE database. Soil Use and Management 25, : 124–127. https://doi.org/10.1111/j.1475-2743.2009.00202.x

Response: We have carefully read all the listed reference and cited the relevant references if necessary.

Reviewers' comments:

Reviewer #3 (Remarks to the Author):

Globally, the authors did a good job for addressing the reviewers concerns. The supplementary analysis are very helpful in understanding the results presented in the manuscript. The uncertainty analysis is now clearly presented and the results adequately discussed and removing the part where the global BRT model was used for making prediction was a good choice. The two answers I still disagree with are the one related to the aggregation of relative importance (RI) of the variables for the 10000 local models and the one about the “inherent” correlation between soil variables and SOC turn over estimates. Working on this latter point, in order to explain clearly why I disagree with authors, I figured out more precisely how calibration and validation datasets where drawn and why the R2 obtained for the global BRT model is so high. I then came to the conclusion that both calibration and validation datasets are only partially independent (contrary to what is written in the text). Hence, the term validation or predictive power should be removed from the text unless a different validation dataset is used (for instance point observations) because it is misleading to write that the proposed model has a validation or prediction R2 of 0.89. I hope that these comments will help to improve the manuscript.

Aggregating the RI over several models :
I fully understand that relative importance is a different concept from the predictive performance and that you may compute mean relative importance of variables over two or more fitted BRT models. However, my concern is about the conclusion one may have when interpreting these averages. Again, what would be the point of averaging RIs of a model with R2=0.05 and those of another one with R2=0.8? I would prefer an analysis saying that, where models had reasonable predictive power, variable A was more important than variable B, hence not trusting models with low predictive power. There are several places in the text where conclusions based on the RI averaging analysis are misleading if not untrue, because model predictive performance is not taken into account. L 223-225 authors write that “climate is more important than soil properties in high northern latitudes highlighting the potential vulnerability of SOC in colder ecosystems such as tundra and boreal forests under climate change.“ and doing so, they make the error of equating change in relative variable importance in BRT models with change of the strength of the relationship between the considered variables, climate ones, and the response, the SOC turn-over. Indeed, model predictive performance is also dropping in these regions, and the rise of RIS of climate variables could be explained by the fact that influence of soil related variables drops while climate influence remains stable, at least equal to what would be observed in other regions of the globe. This is only one possible explanation that can be hypothesized, but it shows why, comparing RI of different models without taking predictive performance (or) fit into account can be misleading.
We may also look jointly at fig. 4B and Supplementary Fig. 4. There, average R2 and RI% of climate clearly seem to be negatively correlated : when average R2 decreases, RI of climate rises, and the “S” pattern mentioned line 121 for RI is also observed for R2 (although mirrored, because of the negative correlation). I do not have the data to quantify the relationship, but at first view this seems to contradict l.126-128 (“It must be noted that the performance of the localized BRT varies with the location of interest, albeit this variation does not affect the results on the relative importance of the assessed variables.”) : variation of the performance of the localized BRT seems somehow related to the results on the relative importance of the assessed variables. It would be more interesting to discuss why, depending on the location, model predictive ability changes (depending on SOC and NPP variability ranges within the local datasets and on the correlation of covariates with SOC and NPP vs. other local scale variables as mentioned briefly l. 132) and take this into account when discussing local RIs.
As a consequence, I think that most of the results (l.108-125) and conclusions (l.216-217, l.221-222, l. 260 for instance) presented in the paper related to the local scale analysis of RIs have little interest as long as models performance are not taken into account. In its current form, the information it brings to the whole paper is not is not significant compared to the results provided

by the global model.

Inherent correlations between soil covariates and response variable :
I appreciated the detail given about the way values of soil related variables are estimated in HWSD : "In brief, soil units in 6 the HWSD are defined based on different soil classification systems (e.g., FAO-90 units) which are independent of soil physiochemical properties [..]. Based on the pre-defined soil units and soil profile measurements, SOC and other soil properties across the globe at the resolution of 1 km were derived using taxonomy-based pedotransfer rules.". 1) I do not understand why, in their response, the authors do claim that soil physiochemical properties are independent from soil units, and 2) please note that the HWSD procedure relies on taxonomy-based pedotransfer rules only for properties for which no data is available within the profiles attached to a given soil unit. Where data is available, soil physiochemical properties are estimated by performing descriptive statistics on measurements made on profiles attached to the soil unit, not by applying pedotransfer rules (see Batjes et al. 2002 for details). In both cases, soil physiochemical properties clearly depend on the soil unit, and reciprocally.
Furthermore, in the dataset used for the present study, where each pixel carries the estimate of the soil unit it belongs to, SOC levels are correlated with other soil variables (let us call them X) and this is a well known result. In turn, as SOC turn over levels are mostly correlated with SOC (fig.2), these soil variables X are also correlated with and may explain SOC turn over levels. This is not necessary a problem and the additional material (such as the new fig. 2) helps to understand the prominent importance of soil variables. Now back to the excellent performance of the BRT model : both the calibration and the validation datasets will share pixels with exactly the same physiochemical values (X, SOC). This is because the probability of drawing at least one pixel from one given soil unit A (except for very small soil units) is not null in the random sampling used for drawing both calibration and validation datasets. Let us take a pixel from calibration dataset and pixel from the validation dataset belonging to the same soil unit. Both pixels will differ only by climate variables and NPP values but will share the same physiochemical values (X, SOC). In turn, unless NPP variability between both pixels greatly impacts computed SOC turn over (but i) as said before NPP has only secondary effect on SOC turn over and ii) climate co-variates help greatly to predict NPP (fig.3, resp. to reviewers)), they will have similar (X, SOC turn-over) pairs. Now, if you generalize, both calibration and validation dataset used for the global BRT model share these similar (X, SOC turn-over) pairs for most of the soil units, and this is why, the BRT model fitted on the calibration dataset is able to very accurately predict on the validation dataset. This explanation applies to the result of the cross-validation results too.
To conclude this point and after thinking about it, it appears that the validation dataset is only partially independent from the validation dataset (because both will contain same (X, SOC) pairs, modulated by NPP to yield similar, or close (X, SOC turn-over) pairs). Providing in the paper "validation results" is misleading to the reader (who might conclude that based on climate and physiochemical variables only the model is able to predict SOC turn over with R2=0.89). That being said, there is still interest in discussing the BRT derived contributions of soil properties vs. climate (landuse should be relevant too) in the proposed SOC turn over estimates. The discussion should include the following items : a) how does the resolution mismatch between NPP data and HWSD data affects the conclusions? b) the SOC turn over is mostly correlated to SOC rather than to NPP hence conclusions reflect mainly the way the database used to estimate SOC (i.e. the HWSD) was build. For point a) above, although resolution of the HWSD raster is 1km, its semantic resolution (the size of the soil mapping units) is much larger, hence the spatial variability of soil properties might not match the one of the modis NPP 1km resolution layers. I would furthermore recommend to remove the validation step of the BRT model (and the related results) and clearly explain that it was fitted to the data in order to understand the relationships between covariates and the response variable, not to provide a model with good predictive performance.

Additional minor comments :
- As mentioned above, the new figure 2 is certainly useful. Does NPP refers to BNPP0-30? If not, it should because this later variable is unused to estimate SOC turn-over, not NPP.

- l.143-147 : when commenting the results of the path analysis, it is unclear what a global negative correlation with climate and a positive correlation with soil are (as in a first place it is assumed that MAT have a positive relationship with SOC turn-over for instance, and soil variable may have positive or negative relationships depending on the variables). Please provide more details here.
- Some soil properties which relative importance in the global model is high may not only be drivers to SOM turn over but also, depending on the pedo-climatic conditions, driven by SOM levels and in turn by SOM turnover rates. This is true for pH or TEB for instance, depending on the litter composition. This is also a well known results for CEC (with is not considered in the present model). This dependence should be mentioned in the paper too.

Responses to reviewers' comments

Reviewer #3 (Remarks to the Author):

Globally, the authors did a good job for addressing the reviewers concerns. The supplementary analysis are very helpful in understanding the results presented in the manuscript. The uncertainty analysis is now clearly presented and the results adequately discussed and removing the part where the global BRT model was used for making prediction was a good choice.

Response: We are grateful for these positive comments on our revision, and thank the reviewer very much for the careful review.

The two answers I still disagree with are the one related to the aggregation of relative importance (RI) of the variables for the 10000 local models and the one about the "inherent" correlation between soil variables and SOC turn over estimates. Working on this latter point, in order to explain clearly why I disagree with authors, I figured out more precisely how calibration and validation datasets where drawn and why the R2 obtained for the global BRT model is so high. I then came to the conclusion that both calibration and validation datasets are only partially independent (contrary to what is written in the text).

Hence, the term validation or predictive power should be removed from the text unless a different validation dataset is used (for instance point observations) because it is misleading to write that the proposed model has a validation or prediction R2 of 0.89. I hope that these comments will help to improve the manuscript.

Response: We appreciate these constructive comments. We especially thank the reviewer for the thorough explanations about her/his concerns and providing detailed recommendations/suggestions to improve the manuscript. According to the comments and suggestions, we have took particularly care to the RI results and the validity of the BRT model validation. For RI results, we have carefully went through the whole manuscript and revised the manuscript to avoid potential misleading interpretation. For model validation and the relevant data independence, we followed the reviewer's suggestions to clarify and discuss the inherent correlations between SOC and other soil physiochemical properties. Please refer to our detailed point-by-point responses below.

Aggregating the RI over several models:

I fully understand that relative importance is a different concept from the predictive performance and that you may compute mean relative importance of variables over two or more fitted BRT models. However, my concern is about the conclusion one may have when interpreting these averages. Again, what would be the point of averaging RIs of a model with R2=0.05 and those of another one with R2=0.8? I would prefer an analysis saying that, where models had reasonable predictive power, variable A was more important than variable B, hence not trusting models with low predictive power. There are several places in the text where conclusions based on the RI averaging analysis are misleading if not untrue, because model predictive performance is not taken into account. L 223-225 authors write that "climate is more important than soil properties

in high northern latitudes highlighting the potential vulnerability of SOC in colder ecosystems such as tundra and boreal forests under climate change." and doing so, they make the error of equating change in relative variable importance in BRT models with change of the strength of the relationship between the considered variables, climate ones, and the response, the SOC turnover. Indeed, model predictive performance is also dropping in these regions, and the rise of RIS of climate variables could be explained by the fact that influence of soil related variables drops while climate influence remains stable, at least equal to what would be observed in other regions of the globe. This is only one possible explanation that can be hypothesized, but it shows why, comparing RI of different models without taking predictive performance (or) fit into account can be misleading.

Response: Thank the reviewer for the comment and the detailed explanation. To address the concern on the effect of model performance on the interpretation of RI, we have conducted another analysis focusing on the importance of climate corrected by model performance (thank the reviewer for her/his suggestion). To do so, we multiplied the RI of climate by the model performance R^2 in each selected 100 * 100 km grid. For example, if the RI of climate is 0.5 and R^2 is 0.8, the corrected RI of climate is 0.4. As R^2 indicated the overall variance explained by the BRT model, this corrected importance reflects the overall importance of climate relative to all potential variables affecting SOC turnover times. Based on the new calculation, we have updated the manuscript including all figures showing the new results (Fig. 1 in this response letter, and Fig. 4 in the revised manuscript) and all relevant texts (e.g., lines 108-114, 468-473 in the revised manuscript). We also calculated the corrected RI of soil (Fig. 1B) and discussed the relative results in the manuscript. Across the globe, the average corrected RI of climate is ~16%, while it is 57% for soil. This result demonstrates the dominant control of soil properties on SOC turnover times at the local scale, although the great variability.

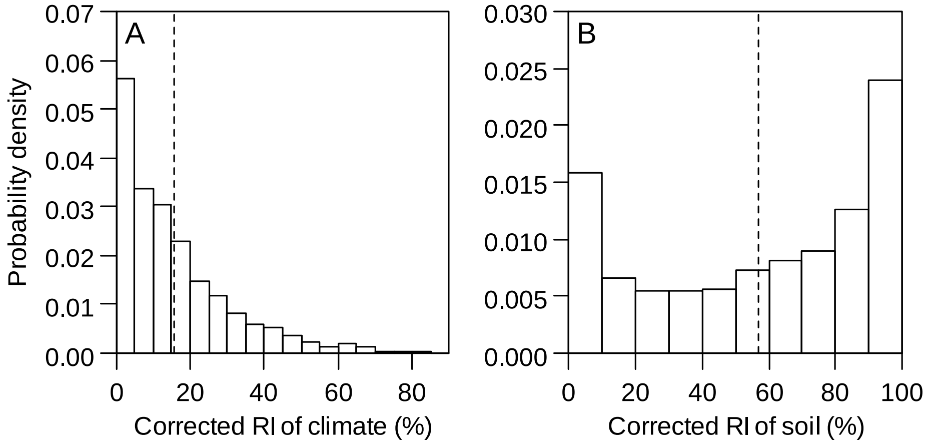

Fig. 1. ***The corrected relative importance (RI) of climate in controlling subsoil SOC turnover.*** *(A) The probability density of the corrected RI of climate. (B) The probability density of the corrected RI of soil. The corrected RI is calculated as the RI predicted by the BRT model multiplied by model performance (i.e.,* R^2*), reflecting the overall importance of climate or soil relative to all potential controls over SOC turnover. The dashed line shows the global average RI (i.e., 16% for climate and 57% for soil).*

We may also look jointly at fig. 4B and Supplementary Fig. 4. There, average R2 and RI% of climate clearly seem to be negatively correlated : when average R2 decreases, RI of climate rises, and the "S" pattern mentioned line 121 for RI is also observed for R2 (although mirrored, because of the negative correlation). I do not have the data to quantify the relationship, but at first view this seems to contradict l.126-128 ("It must be noted that the performance of the localized BRT varies with the location of interest, albeit this variation does not affect the results on the relative importance of the assessed variables.") : variation of the performance of the localized BRT seems somehow related to the results on the relative importance of the assessed variables. It would be more interesting to discuss why, depending on the location, model predictive ability changes (depending on SOC and NPP variability ranges within the local datasets and on the correlation of covariates with SOC and NPP vs. other local scale variables as mentioned briefly l. 132) and take this into account when discussing local RIs.

Response: Thanks for raising this point. To address this point, we explicitly assessed the relationship between R^2 and RI of climate (Fig. 5 in the revised manuscript, Fig. 2 in the next page in this response letter) based on the results for all modelled 100 * 100 km windows rather than the average latitudinal results presented in the Fig. 4B and Supplementary Fig. 4 in last version manuscript. In addition, we used corrected RI taking into account the reviewer's above comment. The results indicated that the points are well below or on the 1:1 line (if the points are on the 1:1 line, climate or soil contributes 100% to the explained variance). In order to detect the average pattern, we first grouped R^2 into 10 groups from 0 to 1 with an increment of 0.1, i.e., [0-0.1), [0.1,0.2), ……, [0.9,1]. For each R^2 group, we calculated average RI of both climate and soil. Then, we assessed the relationship between the average RI and average R^2 in the corresponding R^2 group (i.e., black solid points in Fig. 3). The results indicate that the corrected RI of both climate and soil, and the model performance indicated by R^2 are highly variable as demonstrated in Fig. 2 (i.e., Fig. 4 in the manuscript). In terms of the relationship between average RI and R^2, it is apparent that the corrected average RI of climate has non-linear relationship with model performance, showing a peak of ~28% at the model performance of around 0.5 (Fig. 2A). Lower than this peak, the RI of climate increases and *vice versa*. For the relationship between average RI of soil and model performance, the RI of soil exponentially increases with model performance (Fig. 3B). All these new results have been included in the revised manuscript (see lines 120-133)

Overall, these results demonstrated the variability of the importance of climate and soil in explaining the variability of SOC turnover times at the local scale. We discussed these points and potential underlying causes/consequences in the revised manuscript (see lines 210-230).

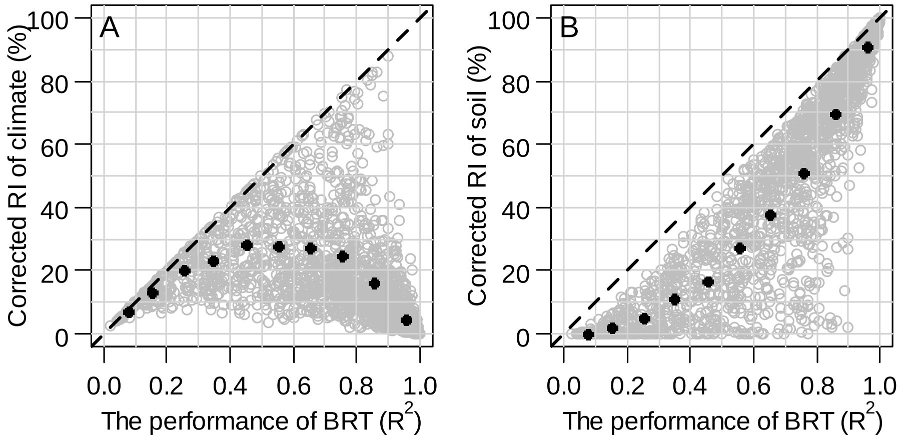

*Fig. 2. The relationship between model performance and the corrected relative importance of climate (A) and soil (B). Grey open circles show the results at each 100 * 100 km window randomly selected for modelling. Black solid circles show the average for each group of R^2 from 0 to 1 with 0.1 increment. The dashed line show the threshold line. See response text for more details.*

As a consequence, I think that most of the results (l.108-125) and conclusions (l.216-217, l.221-222, l. 260 for instance) presented in the paper related to the local scale analysis of RIs have little interest as long as models performance are not taken into account. In its current form, the information it brings to the whole paper is not is not significant compared to the results provided by the global model.

Response: We considered carefully on the results of the local scale analysis taking into account the reviewer's comments (see above responses to the first two points), which actually largely improved the interpretation of the results. We contend to include the local scale results, as the local scale analysis generated an important information of the relative importance of climate and soil for regulating SOC turnover times, and, more importantly, the variability of both RI and R^2 as pointed out by the reviewer. In line with the results at the global scale, the assessment at the local scale demonstrated that soil is much more important than climate for regulating SOC turnover time at the local scale. This information is important for local specific effective carbon management.

Inherent correlations between soil covariates and response variable:

I appreciated the detail given about the way values of soil related variables are estimated in HWSD : "In brief, soil units in 6 the HWSD are defined based on different soil classification systems (e.g., FAO-90 units) which are independent of soil physiochemical properties [..]. Based on the pre-defined soil units and soil profile measurements, SOC and other soil properties across the globe at the resolution of 1 km were derived using taxonomy-based pedotransfer rules.". 1) I do not understand why, in their response, the authors do claim that soil physiochemical properties are independent from soil units, and 2) please note that the HWSD procedure relies on taxonomy-based pedotransfer rules only for properties for which no data is available within the profiles attached to a given soil unit. Where data is available, soil physiochemical properties are

estimated by performing descriptive statistics on measurements made on profiles attached to the soil unit, not by applying pedotransfer rules (see Batjes et al. 2002 for details). In both cases, soil physiochemical properties clearly depend on the soil unit, and reciprocally.

Response: Thank the reviewer for the careful review. We are sorry for that our statement in our last round response is not accurate, which results in some misunderstandings. In our original response, we want to emphasize that soil units "are independent of soil physicochemical properties" rather than the reverse (i.e., soil physiochemical properties are independent from soil units). We had pointed out that soil units are based on FAO classification systems. For the taxonomy-based pedotransfer rules, we have carefully went through the ISRIC report by Batjes 2002 as well as the HSWD technique report. We do agree with the reviewer's comment on the application of taxotransfer rules across the globe. Our initial response incorrectly stated this. In this revision, we have included some descriptions on the approach in lines 307-311. Batjes et al. 2002 is also cited.

Furthermore, in the dataset used for the present study, where each pixel carries the estimate of the soil unit it belongs to, SOC levels are correlated with other soil variables (let us call them X) and this is a well known result. In turn, as SOC turn over levels are mostly correlated with SOC (fig.2), these soil variables X are also correlated with and may explain SOC turn over levels. This is not necessary a problem and the additional material (such as the new fig. 2) helps to understand the prominent importance of soil variables. Now back to the excellent performance of the BRT model : both the calibration and the validation datasets will share pixels with exactly the same physiochemical values (X, SOC). This is because the probability of drawing at least one pixel from one given soil unit A (except for very small soil units) is not null in the random sampling used for drawing both calibration and validation datasets. Let us take a pixel from calibration dataset and pixel from the validation dataset belonging to the same soil unit. Both pixels will differ only by climate variables and NPP values but will share the same physiochemical values (X, SOC). In turn, unless NPP variability between both pixels greatly impacts computed SOC turn over (but i) as said before NPP has only secondary effect on SOC turn over and ii) climate co-variates help greatly to predict NPP (fig.3, resp. to reviewers)), they will have similar (X, SOC turn-over) pairs. Now, if you generalize, both calibration and validation dataset used for the global BRT model share these similar (X, SOC turn-over) pairs for most of the soil units, and this is why, the BRT model fitted on the calibration dataset is able to very accurately predict on the validation dataset. This explanation applies to the result of the cross-validation results too.

Response: We appreciate the detailed explanation on the HWSD database and the estimation of SOC (as well as other soil properties) based on soil units. These detailed explanations make us much clearer on the procedure generating the HWSD database. However, we can do little, if nothing, on the HWSD database itself in this particular study. In this revision, we have provided more details about the HWSD data (see lines 307-311), and also discussed the relevant effects on the results (e.g., lines 216-219, 267-273).

To conclude this point and after thinking about it, it appears that the validation dataset is only partially independent from the validation dataset (because both will contain same (X, SOC) pairs,

modulated by NPP to yield similar, or close (X, SOC turn-over) pairs). Providing in the paper "validation results" is misleading to the reader (who might conclude that based on climate and physiochemical variables only the model is able to predict SOC turn over with R2=0.89). That being said, there is still interest in discussing the BRT derived contributions of soil properties vs. climate (landuse should be relevant too) in the proposed SOC turn over estimates. The discussion should include the following items : a) how does the resolution mismatch between NPP data and HWSD data affects the conclusions? b) the SOC turn over is mostly correlated to SOC rather than to NPP hence conclusions reflect mainly the way the database used to estimate SOC (i.e. the HWSD) was build. For point a) above, although resolution of the HWSD raster is 1km, its semantic resolution (the size of the soil mapping units) is much larger, hence the spatial variability of soil properties might not match the one of the modis NPP 1km resolution layers. I would furthermore recommend to remove the validation step of the BRT model (and the related results) and clearly explain that it was fitted to the data in order to understand the relationships between covariates and the response variable, not to provide a model with good predictive performance.

Response: We are grateful to the reviewer for providing the suggestions and recommendations to address the issue about model validation. We have expanded the discussion on the mismatch between NPP and HWSD data sets by highlighting that HWSD is based on soil units as suggested by the reviewer (see lines 267-273). We also further emphasized the aim of the BRT model in lines 451-453. For the correlation of SOC turnover times with NPP and SOC, we have re-assessed the relationship between SOC turnover times and NPP. In our previous assessment, we used the total NPP rather than the NPP allocated to the 0.3–1 soil layer (i.e., $BNPP_{0.3\text{-}1}$, thank the reviewer very much for pointing out this in her/his first minor comment). In the revision, we have assessed the relationship between SOC turnover times and $BNPP_{0.3\text{-}1}$. The new results suggested that the correlation of SOC turnover times to NPP (r = 0.68) is close to its correlation to SOC (r = - 0.76) although in opposite direction (see the revised Fig. 2 in the manuscript).

We agree with the recommendation on removing the validation steps of the BRT model as the purpose of the BRT modelling is to explain the variance in SOC turnover times rather than providing a model to predict SOC (we included the section of validation because our initial manuscript conducted a prediction of SOC turnover times at climate warming conditions, which has been removed in the last revision). As such, the validation was removed from the manuscript, and the whole manuscript was updated correspondingly.

Additional minor comments:

- As mentioned above, the new figure 2 is certainly useful. Does NPP refers to BNPP0-30? If not, it should because this later variable is unused to estimate SOC turn-over, not NPP.

Response: We appreciate this comment and the careful review. It was total NPP in the last version. In this revision, we used $BNPP_{0.3\text{-}1}$, the figure and relevant results were updated accordingly.

- l.143-147 : when commenting the results of the path analysis, it is unclear what a global negative correlation with climate and a positive correlation with soil are (as in a first place it is

assumed that MAT have a positive relationship with SOC turn-over for instance, and soil variable may have positive or negative relationships depending on the variables). Please provide more details here.

Response: Thanks for the careful review. More details have been supplied with the figure showing the path analysis results in Fig. 6C (lines 677-682). It is also explained in lines 486-490 in the Methods section. The path analysis indicated that climate has negative effect on SOC turnover times. As climate is represented by MAT and MAP while their loading to the latent variable "climate" is positive, that means, as expected, MAT and MAP have negative effect on SOC turnover times. That is, high MAT and MAP may results in low SOC turnover times (but fast turnover). The effect of soil properties can be explained similarly.

- Some soil properties which relative importance in the global model is high may not only be drivers to SOM turn over but also, depending on the pedo-climatic conditions, driven by SOM levels and in turn by SOM turnover rates. This is true for pH or TEB for instance, depending on the litter composition. This is also a well known results for CEC (with is not considered in the present model). This dependence should be mentioned in the paper too.

Response: We appreciate this comment. Yes, all factors potentially correlated to or influencing SOC will affect SOC turnover times as SOC turnover time is calculated by SOC and NPP. We have highlighted this point in lines 444-446.

Response to reviewers' comments

Reviewer #3 (Remarks to the Author):

Thank you to the authors for taking into accounts the previous remarks. Most of the results and conclusions now make sense given the changes that were committed and I now only have few remarks/requests which are minor compared to the previous ones.

Response: Thank the review for her/his careful review and all comments. These comments including those in the last two rounds of reviews have significantly improved this study.

1. The method and results about the variable relative influence are on the right track now. Still I would recommend to standardize averages of Corrected Relative Importance (RIc) for performing local vs. global comparisons, that is when averages on RIc are done :

RIc. = sum_over_i(RIi * R2i) / sum_over_i(R2i)

where i is a given model. That would enable one, I think, to compare comparable quantities (global RI on one side and global averages of RIc on the other side, e.g. lines 216 and 219). This way, averaged RIc of all the variables would sum to 100% as for the initial RI. On the same topic, I disagree with statements on line 115 as RIc do not necessarily represent a % of variance explained by the model (if authors think so then they should demonstrate it). It is a product of a) improvements in squared error risk (RI, Hastie et al. 2001) and b) a % of variance explained by the model.

Response: We appreciate this suggestion. According to the suggestion, in this revision, we calculated weighted averages of RI (RI_w) when performing the local vs. global comparisons and calculating averages across the global as well in the nine biomes. All the relevant statements have been revised with the consideration of this comment. We also revised the statements about the interpretation of RI and Ri_w, and explained them as the relative influence.

Following this, if using these corrected (weighted) RIs is fine when performing averages, looking at them individually as in fig. 4 or fig. 5 is more tedious because the quantities they represent are not straightforward to interpret. To solve that I would:

- Keep corrected RI only for computing mean relative influence of variables.

- Roll back fig. 4 and use RI as it was done in the previous versions of the manuscript (and this would be more consistent with supp. Data where uncorrected RI is still used)

- Change fig. 5 and display RIs as a function of R2 instead of corrected RIs as a function of R2 (which is hard to interpret as corrected RIs equal RI * R2).

That would be enough to yield the needed conclusions, and to take into account the fact that estimated RIs clearly depend on the model performance. Histograms of local RIs should not be

interpreted alone without looking at R2 values associated to RIs as I mentioned .in my previous reviews.

Hastie, T., Tibshirani, R., Friedman, J., 2001. The Elements of Statistical Learning, Data Mining, Inference, and Prediction, Second Edition. Springer Series in Statistics.

Response: We are grateful for the constructive comments, which have greatly improved our manuscript. The recommended three points have been followed in the revision. All relevant tables and figures have been updated based on the suggestions. There is a whole paragraph in the Results section to describe the variability of R^2 at the local scale. There is also a whole paragraph in the Discussion to discuss the relevant potential consequences on the interpretation of our results.

2. The second point relates to the resolution mismatch between climate variables and SOC turn-over estimates. Soil data resolution and spatial definition is better aligned with the resolution of SOC turn-over estimates. Better aligned because SOC data comes from the very same soil map as the other soil variables and because it greatly determines SOC turnover variability. This might be one of the reasons why soil related variables have a predominant influence at the local scale. This explanation is never clearly expressed this way in the text and I think it should be. Nevertheless, All the other limitations are now clearly presented and I agree with one of the major conclusion about the need for improving soil datasets worldwide.

Response: We agree with the reviewer that, ideally, the climate data and SOC turnover estimates should be at the same resolution. In our opinion, as long as the climate can reflect the variability at the local scale, the results on the importance of climate should be valid. In the manuscript, we have discussed the potential effects of resolution mismatch between different data sources on our results (lines 266-272). With the discussion of the relative importance of climate and soil at the local scale (see lines 212-214), we also highlighted that "**This result is reasonable as climate is generally much less variable at the local than at the global scale, while a greater part of soil variability observed at global scale can be observed at a finer scale**".

Some minor remarks :

- line 83-85 : please replace "suggesting […] ." by " as a result of equ. 4 " . This result is not surprising because of the way turn-over is computed.

Response: Replaced accordingly.

- line 132 : replace increase by decrease (if I understand right the plot 5A).

Response: Thanks for the careful review. Replaced accordingly.

- line 143-144 : remove mentions to corrected RI on global fits.

Response: Done.

- line 219 : In ref. 37 local scales refers to maximum extent of hundreds of meter . You cannot use HWSD (resolution > 1e4 meters) to derive conclusions about influence of such a local scale variability of soils. Please replace "while SOC and soil properties exist great spatial variability even at the local scale37"
by "while a greater part of soil variability observed at global scale can be observed at a finer scale".

Response: Thanks for the suggestion. Replaced accordingly.

Line 221: replace "would be" by "is"

Response: Done.

Line 222: replace "is" by "would be"

Response: Done.

Line 223-225 : replace by "The BRT model performance at the local scale, as well as the importance of climate and soil, are highly variable depending on the location. Areas with very low performance imply that ..."

Response: Thanks for the suggestion. Replaced accordingly.

Line 228 : change "the average of corrected RI" in accordance with the point 2 aboveground

Response: Changed accordingly.

Line 445: please replace "to understand the relationships" with "to explore the local variability of the relationships between covariates"

Response: Replaced accordingly.

REVIEWERS' COMMENTS:

Reviewer #3 (Remarks to the Author):

Thank you to the authors for following the various advices. The paper now addresses all the comments and concerns I have had while reviewing it. Some minor remarks :

l.128 & 129 : please replace RIw by RI
l.646-648 : black dots should represent averages of RI instead of RIw. Because these averages are computed within classes of R2, they can be aggregated (contrary to the case where RIs were averaged over the globe and spanned very diverse ranges of R2). The goal of the plot is to show the relationship of RI and R2, not RIw and climate.

Reviewer #3 (Remarks to the Author):

Thank you to the authors for following the various advices. The paper now addresses all the comments and concerns I have had while reviewing it. Some minor remarks :

Response: Thank the reviewer very much for her/his careful review and providing constructive comments to improve the paper.

l.128 & 129 : please replace RIw by RI

Response: Replaced accordingly.

l.646-648 : black dots should represent averages of RI instead of RIw. Because these averages are computed within classes of R2, they can be aggregated (contrary to the case where RIs were averaged over the globe and spanned very diverse ranges of R2). The goal of the plot is to show the relationship of RI and R2, not RIw and climate.

Response: The figure and the relevant statement were updated accordingly.
